# Semaphorin 3E‐Plexin‐D1 Pathway Downstream of the Luteinizing Hormone Surge Regulates Ovulation, Granulosa Cell Luteinization, and Ovarian Angiogenesis in Mice

**DOI:** 10.1002/advs.202417163

**Published:** 2025-05-20

**Authors:** Hanxue Zhang, Jimmy Dhillon, Paul D. Soloway, Bo Shui, Seoyeon Lee, Jennifer K. Grenier, Paul R. Munn, M. Cecilia Ljungberg, Rebecca B Williams, Rainer B. Lanz, Yu‐Hsiang Liao, Yi A. Ren

**Affiliations:** ^1^ Department of Animal Science College of Agriculture and Life Sciences Cornell University Ithaca NY 14853 USA; ^2^ Department of Biomedical Sciences College of Veterinary Medicine Cornell University Ithaca NY 14853 USA; ^3^ Division of Nutritional Sciences College of Agriculture and Life Sciences Cornell University Ithaca NY 14853 USA; ^4^ Genomics Innovation Hub Biotechnology Resource Center Cornell University Ithaca NY 14853 USA; ^5^ Department of Pediatrics Baylor College of Medicine Houston TX 77030 USA; ^6^ Jan and Dan Duncan Neurological Research Institute at Texas Children's Hospital Houston TX 77030 USA; ^7^ Biotechnology Resource Center Imaging Facility Cornell University Ithaca NY 14853 USA; ^8^ Department of Molecular and Cellular Biology Baylor College of Medicine Houston TX 77030 USA

**Keywords:** granulosa cell luteinization, ovarian angiogenesis, preovulatory, Plexin‐D1, Semaphorin 3E

## Abstract

Ovulation is induced by the luteinizing hormone (LH) surge and accompanied by granulosa cell luteinization and ovarian angiogenesis. Semaphorin 3E (Sema3E)‐Plexin‐D1 pathway regulates angiogenesis in other tissues, but its role in the ovary is unknown. Evidence indicates that Sema3E‐Plexin‐D1 pathway plays an important role in the mouse ovary. The expression of Sema3E and its receptor, Plexin‐D1, is dynamically regulated in the mouse ovary downstream of the LH surge. This regulation requires the modulation of chromatin accessibility by CCAAT/enhancer‐binding proteins α and β. Intraovarian injection of recombinant Sema3E results in reduced ovulation, impaired corpus luteum formation, and aberrant ovarian angiogenesis. These in vivo physiological abnormalities are consistent with altered expression of genes regulating these processes, and with data from in vitro cultured granulosa cells and ovarian stromal tissues treated with Sema3E or neutralizing antibody of Plexin‐D1. The findings pinpoint Sema3E‐Plexin‐D1 pathway as a potential therapeutic target for fertility and infertility management.

## Graphical abstract

The Semaphorin 3E (Sema3E)‐Plexin‐D1 pathway mediated by C/EBPα and C/EBPβ downstream of the luteinizing hormone (LH) surge plays important roles in the mouse preovulatory ovary. Timely activation and suppression of this pathway during the preovulatory stage are crucial for ovulation, corpus luteum formation, and proper angiogenesis. The Sema3E‐Plexin‐D1 pathway may serve as a potential new therapeutic target for managing ovulation and luteal function.

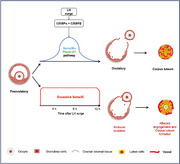

## 1 Introduction

The preovulatory surge of luteinizing hormone (LH) orchestrates intricate cellular and molecular processes in the ovary that culminate in ovulation. In response to the LH surge, preovulatory follicles undergo dynamic cellular changes and tissue remodeling to ensure successful follicle rupture and the subsequent transition into corpus luteum (CL). Follicle rupture and granulosa cell (GC) luteinization are also coordinated with oocyte meiotic maturation, cumulus cell expansion, ovarian angiogenesis, and inflammation of the ovarian tissue. All of these processes must be coordinated with spatial and temporal precision, but the mechanism underlying this coordination remains incompletely understood.

Semaphorin family members are potential novel regulators of preovulatory ovarian function downstream of the LH surge. The semaphorin family contains a number of secreted and membrane‐bound proteins, among which classes 3–7 (Sema3‐7) are expressed in vertebrates.^[^ ^1^ ^]^ In the ovary, Sema4D and Sema4C have been identified as regulators of follicle development and ovarian steroidogenesis.^[^ ^2^ ^]^ Sema6C plays a crucial role in maintaining primordial follicle dormancy and preantral follicle health.^[^ ^3^ ^]^ Additionally, Sema7A is shown to be involved in ovarian follicle remodeling and ovulation.^[^ ^4^ ^]^ An elevated level of transcripts for *Sema3e* in the early luteal phase was reported in mice with GC‐specific double deletion of CCAAT/enhancer‐binding protein α (*Cebpa*) and CCAAT/enhancer‐binding protein β (*Cebpb)*, which have blocked ovulation, defective GC luteinization, and exhibit phenotype suggestive of impaired CL angiogenesis.^[^ ^5^ ^]^ Semaphorin 3E (Sema3E) was initially discovered for its role in axon guidance during neural development.^[^ ^6^ ^]^ However, subsequent research has revealed crucial roles of Sema3E in various tissues beyond the nervous system. For instance, in the cardiovascular system, Sema3E regulates vascular patterning and endothelial cell function.^[^ ^7^ ^]^ Studies have demonstrated that Sema3E, acting through its receptor Plexin‐D1, is critical for maintaining the integrity of the vascular system and preventing abnormal blood vessel formation.^[^ ^8^ ^]^ Additionally, in the immune system, Sema3E modulates the migration and function of immune cells, contributing to immune responses and inflammation.^[^ ^9^ ^]^ Recent research has also indicated that Sema3E can influence tumor progression and metastasis in several cancer types by modulating the tumor microenvironment and angiogenesis.^[^ ^10^ ^]^ These multifaceted roles of Sema3E in different tissues underscore its importance in regulating both physiological and pathological processes. However, the expression and function of the Sema3E‐Plexin‐D1 pathway components in the preovulatory ovary and their involvement in ovulation have not been reported before.

Angiogenesis, the formation of new blood vessels from preexisting ones, is regulated by a balance between pro‐ and anti‐angiogenic factors. Prior to the LH surge, ovarian blood vascular structures are confined to the ovarian stromal tissue (OST), and the GC layers of healthy follicles are devoid of blood vessels. Following the LH surge, GCs express angiogenic regulators that bind to their receptors on endothelial cells (ECs) in OST to regulate their proliferation and migration of these cells, ultimately leading to the formation of an extensive vascular network in the CL. A number of angiogenic regulators have been investigated in depth in the ovary before. Among them, vascular endothelial growth factor A (VEGFA) promotes angiogenesis during follicle development and CL formation.^[^ ^11^ ^]^ Reduced VEGFA expression and impaired VEGFA signaling led to defective ovulation.^[^ ^12^ ^]^ Additionally, injection of Angiopoietin‐2 (ANGPT2) to preovulatory follicles resulted in reduced follicle rupture and prevented the subsequent CL formation.^[^ ^13^ ^]^ Similarly, neutralization of neurotensin via antibody injection into preovulatory follicles disrupts ovulation and proper ovarian vascular remodeling.^[^ ^14^ ^]^ Compromised ovarian angiogenesis is correlated with decreased ovulation rate, along with a reduction in CL size and impaired CL function.^[^ ^15^ ^]^ Together, these findings demonstrate an indispensable role of angiogenesis in ovulation and CL formation/function.

This study aims to investigate the role of the Sema3E‐Plexin‐D1 pathway during ovulation. We hypothesized that the Sema3E‐PlexinD‐1 pathway is activated downstream of the preovulatory LH surge and regulates ovarian function around the time of ovulation. We first characterized the spatial and temporal dynamics of the expression of Sema3E and Plexin‐D1 in the mouse ovary following superovulation. We then tested the function of Sema3E and Plexin‐D1 in the preovulatory ovary in vivo and in vitro. Our data reveal that the Sema3E‐Plexin‐D1 pathway plays an important role in regulating multiple biological processes in the mouse ovary. Our findings provide new mechanistic insights and potential therapeutic targets for female fertility and infertility management.

## 2 Results

### 2.1 Spatial and Temporal Patterns of Expression for *Sema3e* and *Plxnd1* in the Mouse Ovary Around the Time of Ovulation

**Figure 1:**
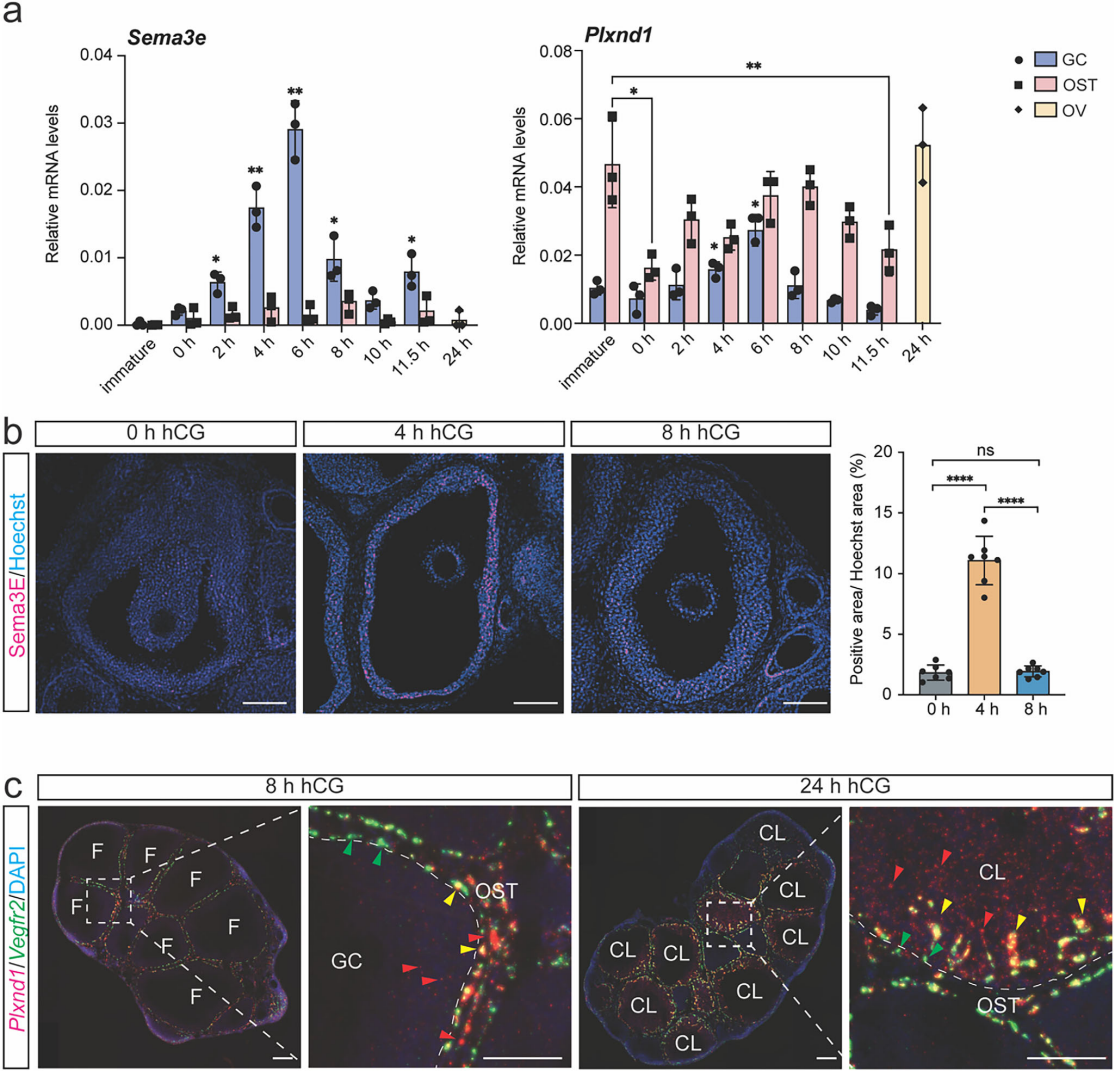
Spatial and temporal patterns of expression for *Sema3e* and *Plxnd1* in the mouse ovary around the time of ovulation. a) RT‐qPCR shows the mRNA levels of *Sema3e* and *Plxnd1* in mouse ovarian GCs (n = 3), OSTs (n = 3), and whole ovaries (n = 3) before and after hCG treatment from superovulated immature mice. b) Representative images of IF staining of Sema3E in mouse ovaries (n = 3 for each time point) before and post‐hCG. The areas of staining for Sema3E were quantified by normalizing the Sema3E‐positive area to the Hoechst‐positive area of preovulatory follicles. Scale bar  =  100 µm. c) Representative images of FISH of *Plxnd1* (red) and *Vegfr2* (green) mRNA in ovaries at 8 h (n = 4) and 24 h (n = 4) post‐hCG. Red arrowheads: *Plxnd1* ^+^ cells. Green arrowheads: *Vegfr2* ^+^ cells. Yellow arrowheads: *Plxnd1* ^+^ *Vegfr2* ^+^ cells. White dashed lines indicate the boundary between the GCs/CL and OST. Scale bar  =  50 µm. Quantitative data are presented as mean ± SD. One‐way ANOVA with Tukey's multiple comparisons test was used for statistical analysis in a (within the same cell/tissue types), b. ^*^ *p* < 0.05; ^**^ *p* < 0.01; ^***^ *p* < 0.001; ns, not significance. GC: granulosa cell; OST: ovarian stromal tissue; OV: whole ovary; F: follicle; CL: corpus lute.

We first examined the expression pattern of *Sema3e* and *Plxnd1* in GCs and OST of the mouse ovary by real‐time quantitative plyomerase chain reaction (RT‐qPCR) (**Figure** **1**a). Superovulation in immature mice was applied to accurately control the timing relative to ovulation. *Sema3e* messenger RNA (mRNA) was induced in GCs as early as 2 h and increased over five‐fold to its maximum level by 6 h post‐human chorionic gonadotropin (hGC). By 24 h post‐hCG, ovulated follicles have formed CL, making it difficult to separate luteinized GCs and OST. Therefore, mRNA from whole ovaries was assessed at 24 h post‐hCG, demonstrating that the levels of *Sema3e* mRNA in whole ovaries had decreased to the basal level as in immature mice. Over the same time course, the induction of *Sema3e* in OST was minimal (Figure 1a, left panel). In contrast, mRNA levels of *Plxnd1* varied across time points in both GCs and the OST, and are higher in the OST than in GCs (Figure 1a, right panel). Notably, in GCs, similar to the pattern of *Sema3e*, the mRNA levels of *Plxnd1* peaked at 6 h post‐hCG; in the OST, mRNA levels of *Plxnd1* decreased after pregnant mare serum gonadotropin (PMSG) stimulation (between immature and 0 h post‐hCG), then increased to a similar level as that at the immature stage shortly after hCG stimulation, remained at a steady level until 11.5 h post‐hCG, and further increased in the whole ovaries at 24 h post‐hCG (Figure 1a, right panel). The spatial and temporal expression dynamics of *Sema3e* and *Plxnd1* were consistent with spatial transcriptomics data (Figure S1a, Supporting Information).^[^ ^16^ ^]^ These findings indicate that mRNA of *Sema3e* and *Plxnd1* is induced by hCG in the mouse ovary with distinct dynamics of expression in GCs versus the OST.

Consistent with the time course RT‐qPCR data, immunofluorescence (IF) staining showed that Sema3E protein was predominantly expressed in GCs (Figure 1b). While minimal expression of Sema3E was detected at 0 h, prominent expression was observed at 4 h and subsequently decreased to the basal level by 8 h post‐hCG. Fluorescent in situ hybridization (FISH) revealed that *Plxnd1* mRNA was robustly expressed in OST at 8 h post‐hCG, with discernible but limited signals in GCs (Figure 1c). *Plxnd1* is shown to be expressed in ECs in various tissues,^[^ ^17^ ^]^ hence we performed co‐FISH of *Plxnd1* with *Vegfr2* (a marker for vascular ECs) in ovaries and found that the majority of *Plxnd1* ^+^ cells within OST were double‐positive of *Plxnd1* and *Vegfr2*. This co‐expression indicates that *Plxnd1* is predominantly expressed by ovarian ECs in OST. Additionally, there were also *Plxnd1* single‐positive cells in the OST, suggesting that it is also expressed by other cell types in this tissue compartment. The cell type‐specific expression of *Sema3e* and *Plxnd1* was further confirmed using publicly available single‐cell RNA seq data (Figure S1b, Supporting Information).^[^ ^18^ ^]^ By 24 h post‐hCG, streams and dispersed *Plxnd1* ^+^ cells, both single‐positive of *Plxnd1* and double‐positive with *Vegfr2*, were observed in abundance in the newly formed CL. In contrast, the number of *Plxnd1* ^+ ^cells in OST appears to have decreased between 8 and 24 h post‐hCG (Figure 1c). These observations suggest the migration of *Plxnd1*‐expressing cells from OST to the newly formed CL after ovulation; they also suggest other cells such as luteal cells express *Plxnd1*. Collectively, these findings demonstrate that the Sema3E‐Plexin‐D1 pathway components are expressed in the ovary in a cell type‐restricted pattern and exhibit dynamic expression both spatially and temporally during ovulation and CL formation. The expression of Sema3E in GCs, together with the expression of Plexin‐D1 in vascular ECs, other stromal cells, and potentially luteal cells suggests that these Plexin‐D1‐expressing cell types are regulated by Sema3E‐Plexn‐D1 pathway in the ovary.

### 2.2 C/EBPα and C/EBPβ‐Modulated Chromatin Accessibility is Essential for the Expression of *Sema3e* and *Plxnd1* Downstream of the LH Surge

**Figure 2:**
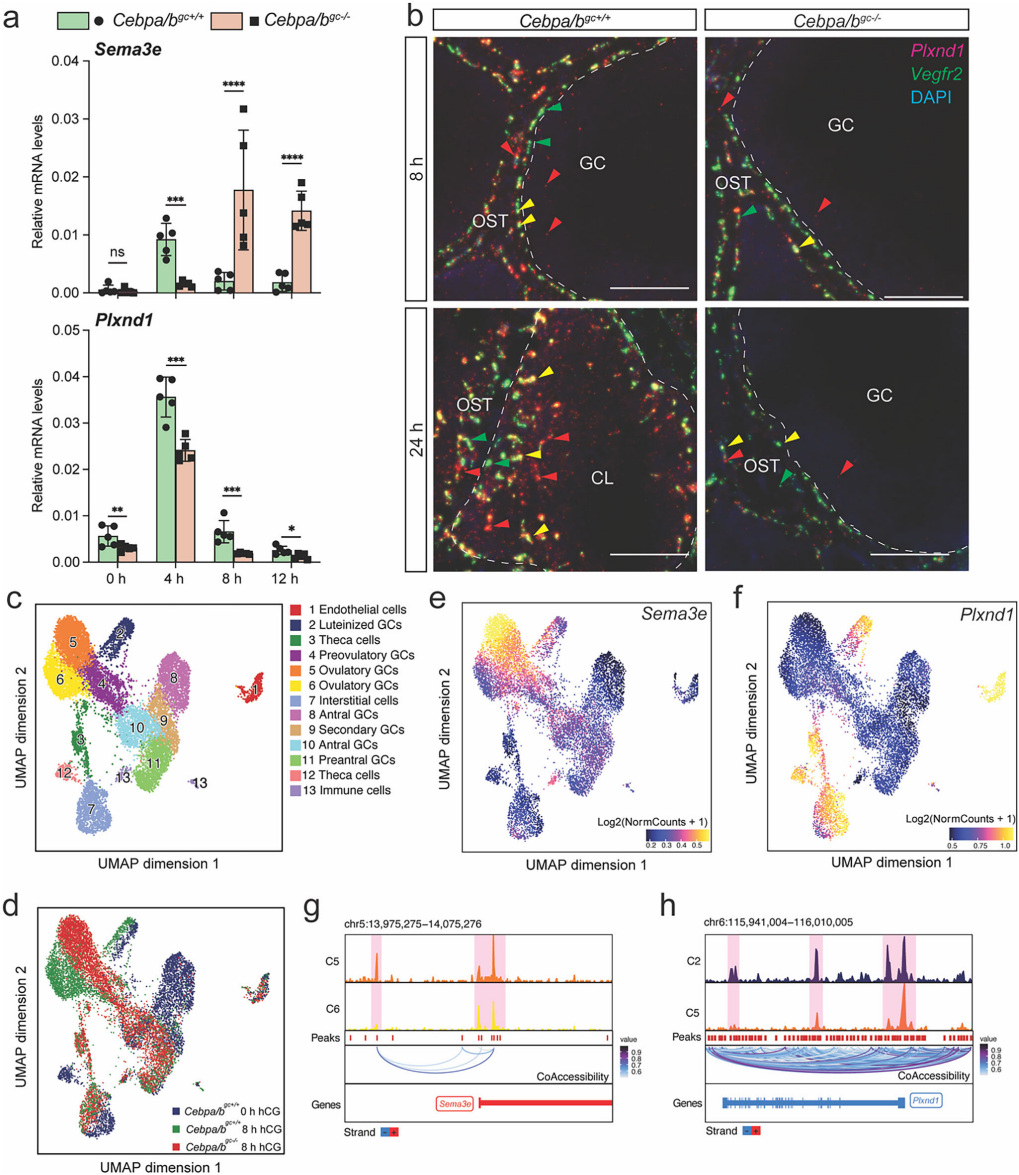
C/EBPα and C/EBPβ‐modulated chromatin accessibility is essential for the expression of *Sema3e* and *Plxnd1* downstream of the LH surge. a) RT‐qPCR shows the mRNA levels of *Sema3e* and *Plxnd1* in GCs of *Cebpa/b^gc+/+^* (n = 5) and *Cebpa/b^gc‐/‐^* mice (n = 5) before and after hCG treatment. b) Representative high magnification images of FISH of *Plxnd1* (red) and *Vegfr2* (green) mRNA in ovaries of *Cebpa/b^gc+/+^* (n = 4) and *Cebpa/b^gc‐/‐^* mice (n = 4) at 8 and 24 h post‐hCG treatment. Red arrowheads: *Plxnd1* ^+^ cells. Green arrowheads: *Vegfr2* ^+^ cells. Yellow arrowheads: *Plxnd1* ^+^ *Vegfr2* ^+^ cells. White dashed lines separate the GCs/CL and OST. Scale bar  =  50 µm. c) UMAP shows clustering and identification of 13 different cell clusters based upon chromatin accessibility patterns in ovaries. d) UMAP shows snATAC‐seq profiles of cell clusters from *Cebpa/b^gc+/+^* controls at 0 and 8 h and *Cebpa/b^gc‐/‐^* mutants at 8 h post‐hCG. Different colors are used to denote different groups (*Cebpa/b^gc+/+^* 0 h: n = 2:  *Cebpa/b^gc+/+^* 8 h: n = 2; *Cebpa/b^gc‐/‐^* 8 h: n = 3). e,f) UMAP visualization colored by log2 normalized gene scores demonstrating the cell cluster‐specific chromatin accessibility in *Sema3e* and *Plxnd1* genes. Gene scores are calculated as Log2(NormCounts + 1). g,h) Genome tracks for the *Sema3e* and *Plxnd1* locus. C2, 5, and 6: cell clusters 2, 5 and 6 in c. Quantitative data are presented as mean ± SD. Multiple two‐tailed unpaired student's test was used for statistical analysis in a. ^*^ *p* < 0.05; ^**^ *p* < 0.01; ^***^ *p* < 0.001; ns, not significance. CL: corpus luteum; F: follicle; GC: granulosa cell; OST: ovarian stromal tissue.

A transgenic mouse line with GC‐specific deletion of *Cebpa* and *Cebpb* (*Cebpa/b^gc‐/‐^*) was reported to have impaired ovulation, CL vascularization, and abnormally increased levels of *Sema3e* mRNA at 24 h post‐hCG.^[^ ^5^ ^]^ We asked whether the expression of *Sema3e* and *Plxnd1* was already abnormal in the ovary of these mutant mice before ovulation, which would suggest that C/EBPα and C/EBPβ (abbreviated as C/EBPα/β hereafter) are required for the activity of this pathway during the preovulatory stage. We compared *Sema3e* and *Plxnd1* mRNA levels in GCs and OST isolated from *Cebpa/b^gc+/+^* control and *Cebpa/b^gc‐/‐^* mutant mice at multiple time points preceding ovulation. Because *Sema3e* mRNA was primarily expressed in GCs of wild‐type (WT) mice (Figure 1a,b), we therefore only assessed *Sema3e* levels in GCs of *Cebpa/b^gc+/+^* controls and *Cebpa/b^gc‐/‐^* mutant mice. Whereas the overall trend of change in the levels of *Sema3e* mRNA in GCs from controls across the four time points was consistent with that in WT mice (**Figure** **2**a upper panel and Figure 1a), mRNA levels of *Sema3e* in GCs of *Cebpa/b^gc‐/‐^* mutants were lower at 4 h post‐hCG but higher from 8 to 12 h post‐hCG compared to controls. Consistent with mRNA levels, Sema3E protein detected by IF staining appeared to be more prominent in *Cebpa/b^gc‐/‐^* mutants compared to controls at 8 h post‐hCG (Figure S2a, Supporting Information). In contrast to *Sema3e*, mRNA levels of *Plxnd1* in GCs were consistently lower in *Cebpa/b^gc‐/‐^* mutants compared to controls at all four‐time points between 0 and 12 h post‐hCG (Figure 2a, lower panel). In OST, *Plxnd1* mRNA levels in *Cebpa/b^gc+/+^* controls rose during the first 8 h post‐hCG and then declined, consistent with observations made in WT mice (Figure S2b, Supporting Information; Figure 1a). In *Cebpa/b^gc‐/‐^* mutants, *Plxnd1* mRNA levels were dysregulated during the first 8 h post‐hCG, showing higher levels relative to controls at 0 and 4 h, and reduced levels at 8 h (Figure S2b, Supporting Information). These observations indicate that C/EBPα/β may differentially regulate the expression of *Sema3e* and *Plxnd1* in GCs and OST before ovulation.

We further applied co‐FISH for *Plxnd1* and *Vegfr2* to evaluate the location and cell types with altered mRNA levels of *Sema3e* and *Plxnd1* in *Cebpa/b^gc‐/‐^* mutants compared to *Cebpa/b^gc+/+^* controls. FISH showed that *Cebpa/b^gc+/+^* controls exhibited a similar pattern of distribution for single‐ and double‐positive cells compared to WT mice at 8 and 24 h post‐hCG (Figure 2b, Figure S2c, Supporting Information; Figure 1c). In *Cebpa/b^gc‐/‐^* mutants, decreased numbers of *Plxnd1* ^+^, *Vegfr2* ^+^, and *Plxnd1* ^+^ *Vegfr2* ^+^ cells were observed in both GCs and OST at 8 h post‐hCG compared to *Cebpa/b^gc+/+^* controls (Figure 2b; Figure S2c,d, Supporting Information). Notably, in contrast to the controls where *Plxnd1* ^+^ *Vegfr2* ^+^ cells were abundant in newly formed CL at 24 h post‐hCG, these cells remained within the OST in *Cebpa/b^gc‐/‐^* mutant at 24 h (Figure 2b; Figure S2c, Supporting Information). These findings indicated that C/EBPα/β expressed by GCs are required for *Plxnd1* expression in both GCs and OST, as well as regulating the migration of *Plxnd1* ^+^ *Vegfr2* ^+^ ovarian vascular ECs.

To probe how C/EBPα/β regulate the expression of *Sema3e* and *Plxnd1* in the ovary, we employed Single‐nucleus Assay for Transposase‐accessible Chromatin sequencing (snATAC‐seq) on whole ovaries obtained from *Cebpa/b^gc+/+^* controls at 0 and 8 h and *Cebpa/b^gc‐/‐^* mutants at 8 h post‐hCG. Unsupervised clustering revealed thirteen major cell clusters that were visualized using Uniform Manifold Approximation and Projection (UMAP) (Figure 2c). We annotated clusters based on differentially accessible genes (Table S1, Supporting Information) and cell type‐specific marker genes known for different ovarian cell types.^[^ ^18^, ^19^ ^]^ Clusters unique to each treatment group (genotype and time point) were visualized by overlaying all three groups after coding each with a different color (Figure 2d). Notably, clusters 2 and 6 (luteinized GCs and ovulatory GCs, respectively) were present in controls but not in *Cebpa/b^gc‐/‐^* mutants at 8 h post‐hCG; in contrast, cluster 5 (ovulatory GCs) was predominant in *Cebpa/b^gc‐/‐^* mutants but not in the controls at 8 h post‐hCG. All three clusters (2, 5, and 6) were not present in controls at 0 h post‐hCG. Additionally, cluster 3 and 12 were identified as theca cells; cluster 12 was present in controls at 0 h post‐hCG, while cluster 3 was present in both control and *Cebpa/b^gc‐/‐^* mutants at 8 h post‐hCG. These observations indicate that hCG induces changes in chromatin accessibility to drive the differentiation of theca cells and at least two sub‐populations of GCs in normal ovulation. However, in the absence of *Cebpa/b*, the differentiation process in GCs is disrupted, leading to aberrant GC fate.

We next examined cell cluster‐specific chromatin accessibility of *Sema3e* and *Plxnd1* and compared it between *Cebpa/b^gc+/+^* controls and *Cebpa/b^gc‐/‐^* mutants (Figure 2c–f). In controls, all clusters present at 0 h post‐hCG had low chromatin accessibility of *Sema3e*; and clusters 2 and 6, which arose at 8 h post‐hCG, had low‐to‐moderate chromatin accessibility. Strikingly, ovulatory GCs of *Cebpa/b^gc‐/‐^* mutants (cluster 5) had significantly elevated chromatin accessibility of *Sema3e* compared to the ovulatory GCs from *Cebpa/b^gc+/+^* controls (cluster 6) at 8 h post‐hCG. Browser plot of peaks confirmed elevated chromatin accessibility in the promoter and putative enhancer regions for *Sema3e* in cluster 5 compared to cluster 6 (Figure 2g). These patterns of chromatin accessibility run parallel to the changes in mRNA levels of *Sema3e*: in WT and *Cebpa/b^gc+/+^* control mice, they were induced by hCG treatment and decreased to near‐basal levels by 8 h post‐hCG (Figures 1a,b and 2a), while in *Cebpa/b^gc‐/‐^* mutants, they were significantly elevated compared to controls at 8 h post‐hCG and sustained at the elevated level till at least 12 h post‐hCG (Figure 2a; Figure S2a, Supporting Information). These observations suggest that C/EBPα and C/EBPβ may actively suppress the expression of *Sema3e* between 4 and 8 h post‐hCG by reducing its chromatin accessibility.

Different from the restricted chromatin accessibility of *Sema3e*, *Plxnd1* exhibited high chromatin accessibility in ECs, theca cells, and sub‐populations of interstitial cells in both *Cebpa/b^gc+/+^* controls and *Cebpa/b^gc‐/‐^* mutants at 0 and 8 h post‐hCG (Figure 2c,d,f). However, cluster 2 (luteinized GCs), which have high chromatin accessibility of *Plxnd1* in controls, was absent in *Cebpa/b^gc‐/‐^* mutants at both time points. Consistently, browser plot of peaks in cluster 5 and cluster 2 confirmed reduced accessibility in the promoter and gene body for *Plxnd1* in *Cebpa/b^gc‐/‐^* mutants (Figure 2h). This result aligned with the lower mRNA levels of *Plxnd1* in GCs of *Cebpa/b^gc‐/‐^* mutants (Figure 2a). These observations indicate that C/EBPα/β modulate chromatin accessibility whereby influencing the expression of *Sema3e* and *Plxnd1* in GCs during the preovulatory stage.

### 2.3 Excessive Sema3E Impairs Ovulation and Affects Ovarian Vasculature

**Figure 3:**
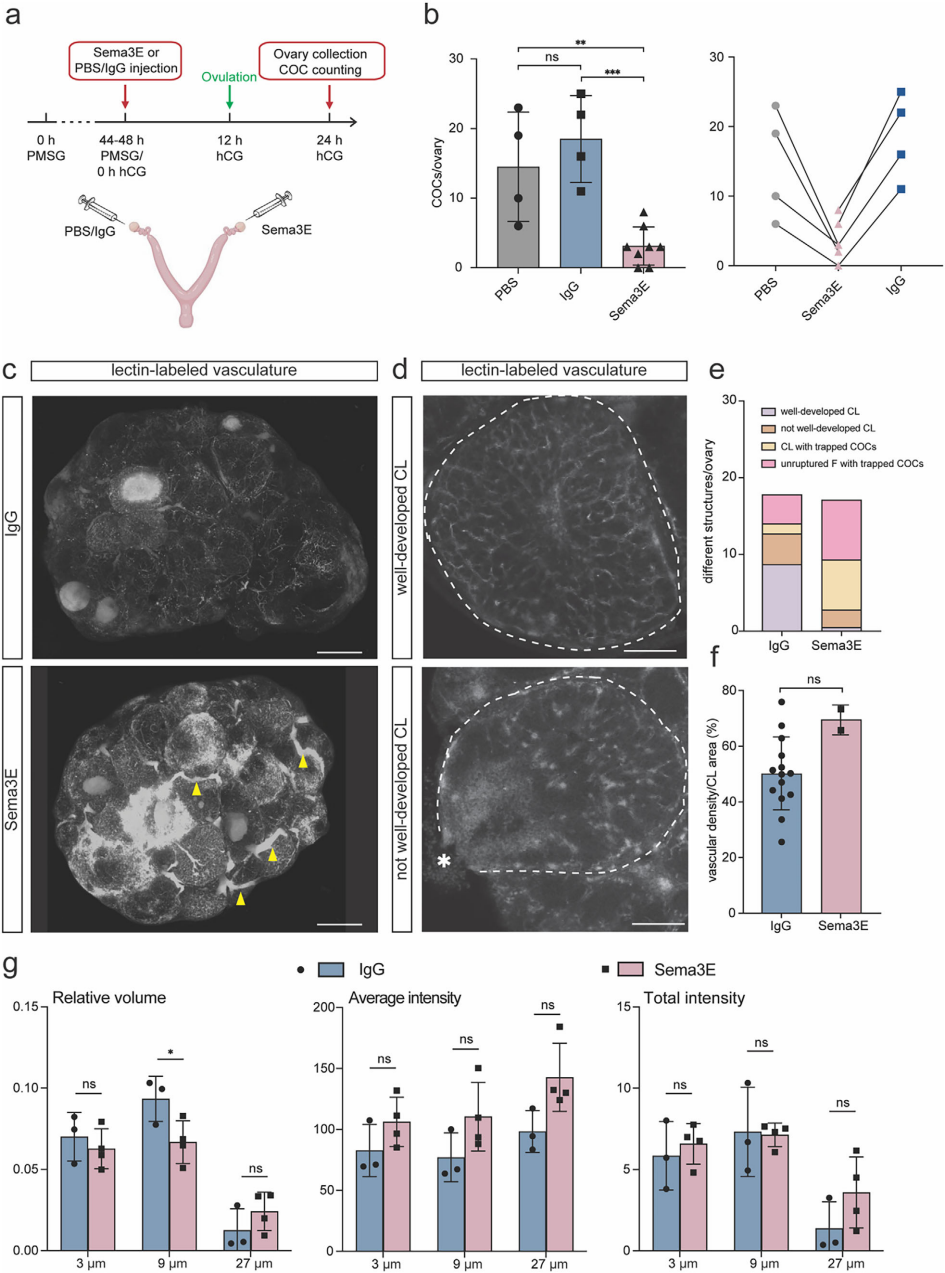
Excessive Sema3E impairs ovulation and affects ovarian vasculature. a) Schematic illustration of the experimental design for intraovarian injection of Sema3E. b) The numbers of ovulated COCs from Sema3E‐ (n = 8), IgG‐ (n = 4), and PBS‐ (n = 4) injected ovaries, respectively. Linked dots represent data from the two ovaries of the same mice. c) Representative whole‐mount 3D projection images of cleared ovaries with blood vessels labeled by lectin conjugated with Alexa 649 (white) at 24 h post‐hCG. Yellow arrowhead: abnormal large blood vessels in Sema3E‐injected ovaries that are not observed in IgG‐injected ovaries. Scale bar  =  500 µm. n = 3 for IgG‐injected ovary. n = 4 for Sema3E‐injected ovary. d) Representative cross‐section images of distinct CL structures in c. Top: well‐developed CL; bottom: not well‐developed CL. CL is outlined by white dashed lines. Asterisk: follicle rupture site that remains open. Scale bar  =  100 µm. e) Proportion of distinct F and CL structures in IgG‐ versus Sema3E‐injected ovaries in c. f) Vascular density of well‐developed CL structures in IgG‐ versus Sema3E‐injected ovaries in c. Vascular density was quantified by normalizing the lectin‐positive area to CL area. g) Hessian tubeness analysis of vascular properties based on whole‐mount imaging of lectin‐labeled blood vessel structures in IgG‐ versus Sema3E‐injected ovaries in c. Quantitative data are presented as mean ± SD. One‐way ANOVA with Tukey's multiple comparisons test was used for statistical analysis in b. Two‐tailed unpaired student's test was used for statistical analysis in f. Multiple two‐tailed unpaired student's test was used for statistical analysis in g. ^*^ *p* < 0.05; ^**^ *p* < 0.01; ^***^ *p* < 0.001; ns, not significance. COC: cumulus‐oocyte complex; CL: corpus luteum; F: follicle.

Observations shown in Figure 2 suggest that the expression of *Sema3e* is actively suppressed at least between 8 to 12 h post‐hCG in normal conditions, and is abnormally upregulated in the absence of C/EBPα/β. To explore the functional impact of elevated Sema3E in the mouse ovary, we performed intraovarian injection of 0.2 µg µL^−1^ mouse recombinant Sema3E before or after hCG stimulation during standard superovulation. As illustrated in **Figure** **3**a, one ovary was treated with Sema3E while the contralateral ovary was treated with phosphate‐buffered saline (PBS) or mouse immunoglobulin G (IgG). Ovulation rates were assessed by counting the number of cumulus‐oocyte‐complexes (COCs) in the oviducts at 24 h post‐hCG (12 h post‐ovulation). When administered right before hCG (0 h post‐hCG), intraovarian injection of Sema3E led to a significant reduction in ovulation rate as compared to the injection of PBS or IgG (Figure 3b). To further delineate the time frame of action by Sema3E relative to ovulation, we also tested the effects of recombinant Sema3E injected at 8 h post‐hCG, which led to a slight but not significant decrease in ovulation rates compared with PBS group (Figure S3a, Supporting Information). These results demonstrate that excessive Sema3E during the preovulatory period leads to a reduced rate of ovulation, and this effect primarily occurs during the early‐ to mid‐preovulatory stage post‐hCG (0 to 8 h). It is probable that excessive Sema3E expression in the *Cebpa/b^gc‐/‐^* mutants contributes to their ovulation defects.

Based on the function of Sema3E in regulating angiogenesis in other tissues, we then assessed whether excessive Sema3E would have an impact on ovarian vasculature around the time of ovulation. We collected ovaries with vascular structures labeled by Alexa 649‐conjugated lectin at 24 h post‐hCG andfrom treatments (Sema3E versus IgG) and performed whole‐mount imaging (Figure 3c). We observed distinct disorganization of vascular structure in the Sema3E‐treated group when compared to the control group. Specifically, the Sema3E‐treated group displayed abnormal, enlarged vessels surrounding follicles and CL across the ovary, and these abnormal structures were not observed in the control group. These data indicated that excessive Sema3E led to altered vascular structure in OST.

We next examined the effect of excessive Sema3E on angiogenesis and the development of CL at 24 h post‐hCG. At this time point and in a normal condition, early CL form with vigorous angiogenesis from the ovarian stroma into the luteinized GC layer. We first calculated the numbers of distinct CL and follicle structures in IgG‐ and Sema3E‐injected ovaries using whole‐mount imaging. CL without a trapped COC and with a closed rupture site were identified as well‐developed CL; in contrast, CL with an open rupture site were categorized as not well‐developed CL (Figure 3d). Among structures containing COCs, those with a large fluid‐filled antrum and limited vascular structure were classified as unruptured follicles with trapped COC, while those filled with cells, containing minimal fluid, and exhibiting a dense vasculature structure were classified as CL with trapped COC (Figure S3b, Supporting Information). Consistent with a reduced rate of ovulation, Sema3E‐treated groups had more unruptured follicles and CL with trapped COCs (Figure 3e). In addition, among the CL without trapped COCs, the Sema3E‐treated group had significantly lower proportions of well‐developed CL compared to the control group. Unlike the well‐developed CL in the control group with closed rupture sites, the majority of CL in the Sema3E‐treated group were not well‐developed and retained an open rupture site with minimal lectin‐labeled capillaries or had trapped COCs (Figure 3d,e; Figure S3b, Supporting Information), suggesting delayed ovulation and/or compromised angiogenesis in the early CL. Interestingly, in well‐developed CL, quantification of vascular density revealed that Sema3E treatment resulted in a trend of increase in vascular density as compared to controls (Figure 3f). We further quantified features of vascular structures across the whole ovary using various algorithms of Hessian Tubeness analysis, which are capable of identifying, classifying, and quantifying features of tubular structures.^[^ ^20^ ^]^ This analysis revealed that compared to IgG‐injected controls, intraovarian injection of Sema3E resulted in *i*) a reduction in the relative volume of blood vessels close to a diameter of 9 µm; *ii*) a trend toward increased average intensity of blood vessels across all sizes, in particular, those close to a diameter of 27 µm, which is consistent with visibly more larger blood vessels (Figure 3c; Figure S3c, Supporting Information); and *iii)* similar total intensity and dimensionless surface area to volume ratio (DSAV) of blood vessels across all sizes (Figure 3g; Figure S3d, Supporting Information). These findings suggest that Sema3E injection deceased the presence of 9 µm vascular structures and induced a trending increase in the presence of 27 µm vascular structures, while having no impact on the overall complexity of vascular structures in the ovary. These results collectively demonstrate that intraovarian injection of Sema3E led to decreased ovulation rates, disrupted CL formation, and altered angiogenesis in both OST and newly formed CL.

### 2.4 Excessive Sema3E Leads to Altered mRNA Levels of Genes Involved in Luteinization, Angiogenesis, and Inflammation

**Figure 4:**
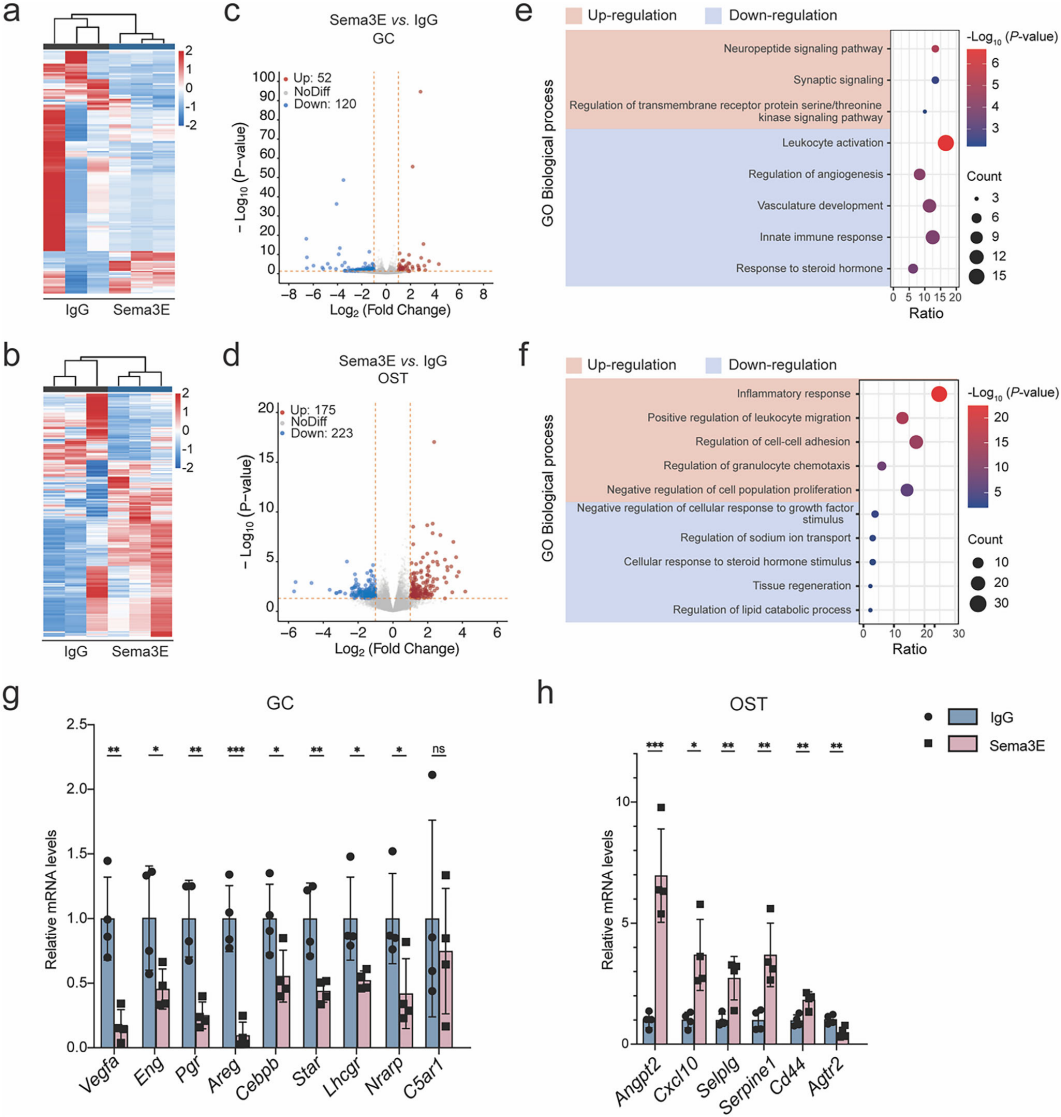
Excessive Sema3E leads to altered mRNA levels of genes involved in luteinization, angiogenesis, and inflammation. a,b) Heatmaps show the z‐score of DEGs in GCs (a) and OSTs (b) from IgG‐ (n = 3) and Sema3E‐injected ovaries (n = 3) at 8 h post‐hCG, respectively. Absolute Fold Change > 2 and *P*‐value < 0.05. c,d) volcano plots show the DEGs in GCs (c) and OSTs (d) from Sema3E‐injected ovaries compared to those from IgG‐injected ovaries. e,f) GO analysis of DEGs in (a) GCs and (b) OSTs (*P*‐value < 0.05). g) RT‐qPCR shows the mRNA expression levels of selected genes in GC of IgG‐ (n = 4) and Sema3E‐injected mice (n = 4) at 8 h post‐hCG. h) RT‐qPCR shows the mRNA expression levels of selected genes in OSTs of IgG‐ (n = 4) and Sema3E‐injected mice (n = 4) at 8 h post‐hCG. Quantitative data are presented as mean ± SD. Two‐tailed unpaired student's test was used for statistical analysis in g, h.  ^*^ *p* < 0.05; ^**^ *p* < 0.01; ^***^ *p* < 0.001; ns, not significance. GC: granulosa cell; OST: ovarian stromal tissue.

To unbiasedly probe the molecular and cellular mechanism underlying disrupted ovulation and ovarian vasculature caused by excessive Sema3E, we isolated GCs and OST at 8 h post‐hCG from ovaries injected with IgG versus Sema3E and performed bulk RNA‐sequencing (RNA‐seq) analysis. Principal component analysis demonstrated that the transcriptomes of the Sema3E‐injected GCs and OST were distinct from that of the IgG‐injected groups (Figure S4, Supporting Information). Heatmap of differentially expressed genes (DEGs) showed that transcriptomic profiles of GCs and OST were substantially altered in Sema3E groups compared to those in IgG groups (**Figure** **4**a,b). A total of 172 DEGs were detected in GCs and 398 DEGs were detected in the OST of the Sema3E group compared to the IgG group. Among these, 52 genes were up‐regulated and 120 down‐regulated in GCs, and 175 up‐regulated and 223 down‐regulated in OST (Figure 4c,d). Gene Ontology (GO) analysis of up‐regulated DEGs in GCs showed that these DEGs are primarily involved in neuropeptide and synaptic signaling pathways, which are consistent with known function of Sema3E‐PlexinD‐1 pathway in regulating neuronal growth and axon guidance.^[^ ^21^ ^]^ Among the down‐regulated DEGs, GO analysis revealed that regulation of angiogenesis and vasculature development were affected in the Sema3E group (Figure 4e), in line with the abnormal ovarian vasculature described above (Figure 3c). The Sema3E group also showed down‐regulation of genes involved in leukocyte activation and innate immune response, as well as genes regulating responses to steroid hormones (Figure 4e). In OST, the up‐regulated DEGs are involved in inflammatory response, immune cell trafficking, cell‐cell adhesion, and negative regulation of cell proliferation (Figure 4f). Among down‐regulated DEGs in OST, genes involved in cellular responses to steroid hormones echoed those of the same GO category among down‐regulated DEGs in GCs.

We further compared the relative mRNA levels of selected DEGs classified into major biological processes described above between IgG‐ versus Sema3E‐treated groups in GCs and OST using RT‐qPCR (Figure 4g,h). Consistent with the bulk RNA‐seq data (Table S2, Supporting Information), genes with significantly reduced transcript levels in GCs of the Sema3E group compared to controls included angiogenic regulators (*Vegfa*, *Eng*, and *Nrarp*),^[^ ^22^ ^]^ genes regulating steroid hormone response, ovulation, and luteinization (*Pgr*, *Areg*, *Cebpb*, *Star*, and *Lhcgr*)^[^ ^5^, ^19^, ^23^ ^]^ (Figure 4g). In OST, transcript levels of immune and inflammation‐related genes (*Anpgt2*, *Cxcl10*, *Selplg*, and *Serpine1*)^[^ ^24^ ^]^ and gene associated with cell surface adhesion (*Cd44*)^[^ ^25^ ^]^ were increased, while the transcript level of steroid response and ovulation related gene, *Agtr2*,^[^ ^26^ ^]^ was decreased in the Sema3E group (Figure 4h). Taken together, these results indicate that excessive Sema3E in vivo affects ovarian response to steroid hormones, luteinization and angiogenic function of GC, as well as inflammation, response to steroid hormone stimulus, and cell‐cell adhesion in OST in vivo.

### 2.5 Crosstalk Between Granulosa Cells and the Ovarian Stromal Tissue is Crucial for Stromal Angiogenesis and Inflammation Downstream of Sema3E‐Plexin‐D1 Pathway

**Figure 5:**
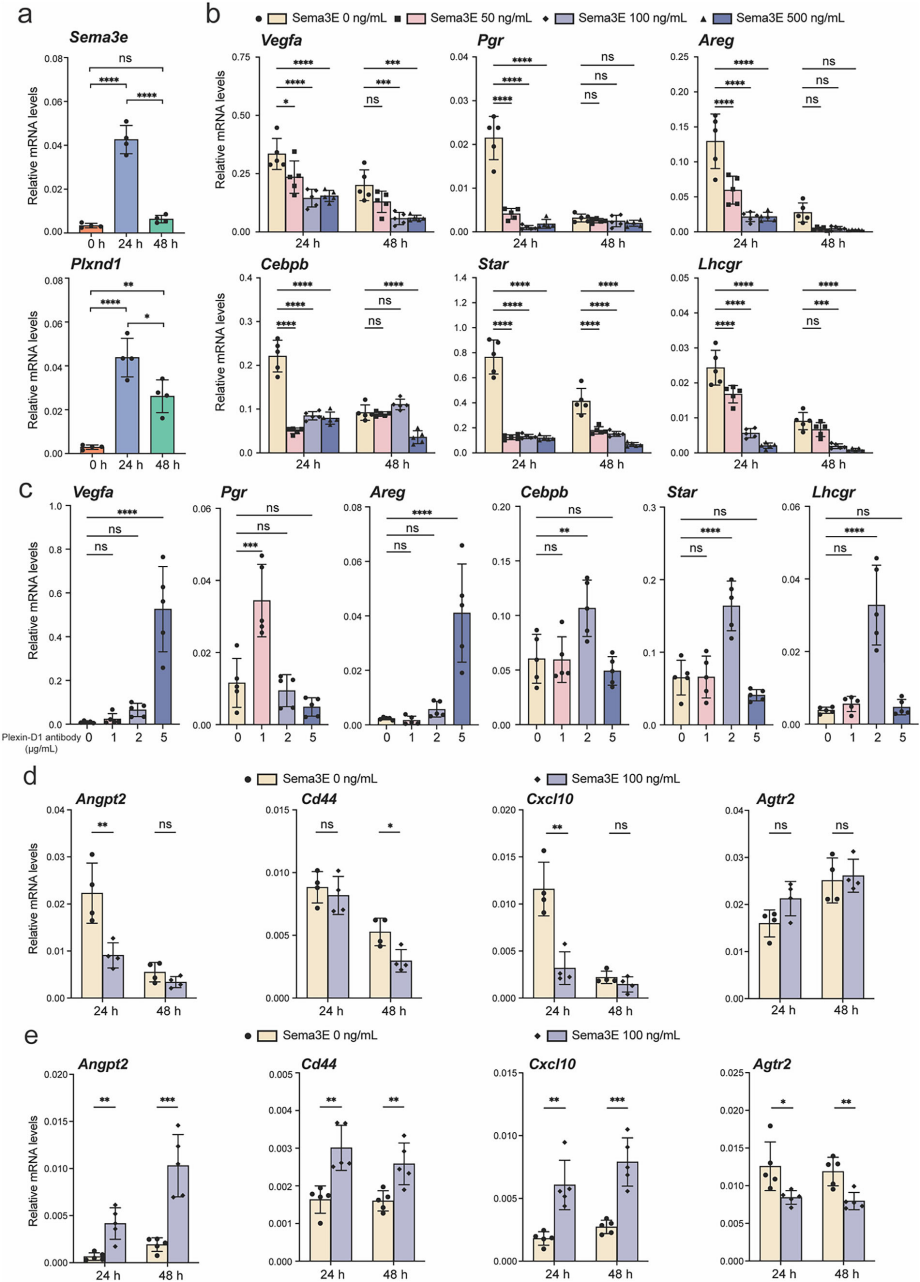
Crosstalk between granulosa cells and ovarian stromal tissue is crucial for stromal angiogenesis and inflammation downstream of Sema3E‐Plexin‐D1 pathway. a) RT‐qPCR shows the induction of *Sema3e* and *Plxnd1* mRNA in in vitro cultured GCs (n = 4) following For/PMA‐induced luteinization. b) RT‐qPCR shows the mRNA levels of selected genes in cultured GCs (n = 5) treated with different concentrations of Sema3E following For/PMA‐induced luteinization. c) RT‐qPCR shows the mRNA expression levels of selected genes in in vitro cultured GCs (n = 5) that were treated with various concentrations of Plexin‐D1 neutralizing antibody at 24 h post‐For/PMA‐induced luteinization. d) RT‐qPCR shows the mRNA expression levels of selected genes in in vitro cultured OSTs (n = 4) that were treated with For/PMA with or without recombinant Sema3E. e) RT‐qPCR shows the mRNA expression levels of selected genes in OSTs (n = 5), which were co‐cultured with GCs and treated with For/PMA with or without recombinant Sema3E. Quantitative data are presented as mean ± SD. One‐way ANOVA with Tukey's multiple comparisons test was used for statistical analysis in a, b. Multiple two‐tailed unpaired student's test was used for statistical analysis in c, d. ^*^ *p* < 0.05; ^**^ *p* < 0.01; ^***^ *p* < 0.001; ns, not significance. GC: granulosa cell; OST: ovarian stromal tissue. For/PMA: forskolin and phorbol 12‐myristate 13‐acetate.

To investigate the direct impact of Sema3E on GCs and OST, respectively, we performed in vitro culture of GCs and OST treated with or without Sema3E. First, to test whether the expression of *Sema3e* and *Plxnd1* is induced in the in vitro culture system during GC luteinization, we treated the cultured GCs with forskolin and phorbol 12‐myristate 13‐acetate (For/PMA), which were previously used to induce luteinization (Figure S5a, Supporting Information).^[^ ^27^ ^]^ As expected for in vitro GC luteinization, which occurs slower than that in vivo, the mRNA of both *Sema3e* and *Plxnd1* were induced at 24 h after For/PMA treatment (**Figure** **5**a). Subsequently, *Sema3e* mRNA returned to the basal level at 48 h post‐treatment, while *Plxnd1* mRNA levels decreased relative to 24 h but remained higher than 0 h. Next, we treated cultured GCs with a series of concentrations of Sema3E to determine dose‐dependent effects and the optimal concentration of Sema3E to be used in the culturing system. Consistent with in vivo data, in vitro cultured GCs exhibited reduced mRNA levels of *Vegfa*, *Pgr*, *Areg*, *Cebpb*, *Star*, and *Lhcgr* after Sema3E treatment, with the mRNA levels of *Vegfa*, *Areg*, and *Lhcgr* exhibiting a dose‐dependent reduction at 24 h post‐luteinization induction (Figure 5b). This aligns with previous published data indicating that Sema3E had a dose‐dependent inhibitory effect on gene expression.^[^ ^28^ ^]^ Most genes we tested showed no dose‐dependent response when treated with Sema3E at 100 ng mL^−1^ versus 500 ng mL^−1^, and the inhibitory effects on gene expression were most prominent at 100 ng mL^−1^; therefore, we used this concentration for subsequent culturing experiments. In addition to mRNA levels, the protein levels of *Vegfa* and *Cebpb* were also decreased in the Sema3E‐treated group (Figure S5b, Supporting Information). To test the direct biological impact of inhibiting Sema3E‐Plexin‐D1 pathway activity on GCs, we treated the cultured GCs with a Plexin‐D1 neutralizing antibody which functions to counteract the binding of Sema3E.^[^ ^29^ ^]^ Opposite to Sema3E treatment, treatment of Plexin‐D1 neutralizing antibody on GCs led to increased mRNA levels of *Vegfa*, *Pgr*, *Areg*, *Cebpb*, *Star*, and *Lhcgr* (Figure 5c), with varied magnitude of induction depending on the concentration employed. To further confirm that Plexin‐D1 is the functional receptor of Sema3E in the ovary, we pre‐treated the cultured GCs with Plexin‐D1 neutralizing antibody 1 h before administering Sema3E. Plexin‐D1 neutralizing antibody effectively blocked the inhibitory effect of Sema3E on the expression of *Vegfa*, *Cebpb*, *Lhcgr*, and *Areg* (Figure S5c, Supporting Information). These data indicate a direct effect of Sema3E‐Plexin‐D1 pathway on the expression of genes regulating ovulation, luteinization, and angiogenesis in the GCs.

In contrast to GCs, in vitro cultured OSTs treated with Sema3E did not mirror the same trend of changes in mRNA levels (*Angpt2*, *Cd44*, *Cxcl10*, and *Agtr2*) as observed in vivo (Figures 4h and 5d). These results suggest that OST may function differently in vivo versus in vitro, and/or that some of its in vivo functions rely on crosstalk with GCs. Indeed, while mRNA levels of angiogenesis‐related genes (*Plxnd1*, *Vegfr2* and *Angpt2*) were increased by hCG treatment in vivo (Figure S5d, Supporting Information), they were unchanged (*Plxnd1*) or reduced (*Vegfr2* and *Angpt2*) in cultured OSTs after For/PMA treatment (Figure S5e, Supporting Information). To test whether GCs‐OST crosstalk mediates the function of OST, we co‐cultured OSTs with GCs with or without Sema3E during For/PMA‐induced luteinization. Co‐culturing with GCs resulted in an increase in the mRNA levels of angiogenic‐related genes (*Plxnd1* and *Vegfr2*) in OST following For/PMA treatment (Figure S5f, Supporting Information). Furthermore, when co‐cultured with GCs, Sema3E treatment altered mRNA levels of *Angpt2*, *Cd44*, *Cxcl10*, and *Agtr2* in OST in patterns mirroring those observed in vivo (Figures 4h and 5e). These results support the idea that crosstalk between GCs and OST is crucial in mediating the effect of Sema3E on OST, underscoring the indispensable role of GCs in regulating OST angiogenesis and inflammation downstream of Sema3E. Taken together, these findings suggest that Sema3E directly regulates GC (ovulation, luteinization, and angiogenesis) but indirectly regulates OST (angiogenesis, inflammation, and cell‐cell adhesion) via its primacy on GCs.

## 3 Discussion

We provide several lines of evidence supporting a role of the Sema3E‐PlexinD‐1 axis in ovarian responses to the preovulatory LH surge that are vital for fertility. These include their spatial and temporal patterns of expression during ovulation; that their manipulation impairs ovulation, ovarian vascular remodeling, and expression of genes important to luteinization; and that the Sema3E‐PlexinD‐1 axis mediates crosstalk between OST and GCs. Additionally, we show that expression and chromatin accessibility of *Sema3e* and *Plxnd1* are both impaired in GCs of mice lacking *Cebpa* and *Cebpb*, which are defective in ovulation, GC luteinization, and CL vascularization.

To our knowledge, there is little prior information on whether and how *Sema3e* or *Plxnd1* are expressed in the mouse ovary, particularly during the preovulatory‐ovulatory stage (0‐12 h post‐hCG) and early luteal phase (up to 24 h post‐hCG). We show that *Sema3e* expression is induced by the LH surge in two sub‐populations but not all ovulatory GCs at 8 h post‐hCG. In both populations, namely ovulatory GCs and luteinized GCs, while only low‐to‐moderate levels of chromatin accessibility of *Sema3e* is observed in normal controls, *Cebpa/b^gc‐/‐^* mutant mice exhibit high chromatin accessibility of *Sema3e* in ovulatory GCs (Figure 2e). These data support that between 8–12 h post‐hCG, C/EBPα/β appear to be indispensable for actively suppressing transcription of *Sema3e*. In contrast, between 0–4 h post‐hCG, C/EBPα/β appear to be required for the induction of *Sema3e* during this early preovulatory stage (Figure 2a). These observations support a biphasic mode of regulation of *Sema3e* transcription in GCs by C/EBPα/β downstream of the LH surge: induction between 0–4 h and suppression between 8–12 h post‐hCG. The mechanism underlying this regulation is likely to be timing‐dependent, with C/EBPα/β exerting opposing effects at different preovulatory stages. This may be mediated through interactions with different transcription factors and transcriptional coactivators,^[^ ^30^ ^]^ but other mechanisms may also be accountable. Given that C/EBPα/β play a crucial role in global chromatin accessibility changes during GC differentiation following hCG/LH stimulation (Figure 2c,d), they may also influence *Sema3e* expression by modulating chromatin accessibility, a possibility warrants further investigation. Furthermore, the in vivo and in vitro effects of Sema3E treatment demonstrate that Sema3E reduced the transcript levels of *Cebpb*, whereas blocking Sema3E‐Plexin‐D1 signaling using the Plexin‐D1 neutralizing antibody increased *Cebpb* expression (Figures 4g and 5b,c; Figure S5c, Supporting Information). Taken together, these findings suggest that Sema3E, induced during early preovulatory stage, negatively feedbacks to suppress the expression of *Cebpb*, forming a regulatory feedback loop between the *Sema3e* and *Cebpb*.

**Figure 6:**
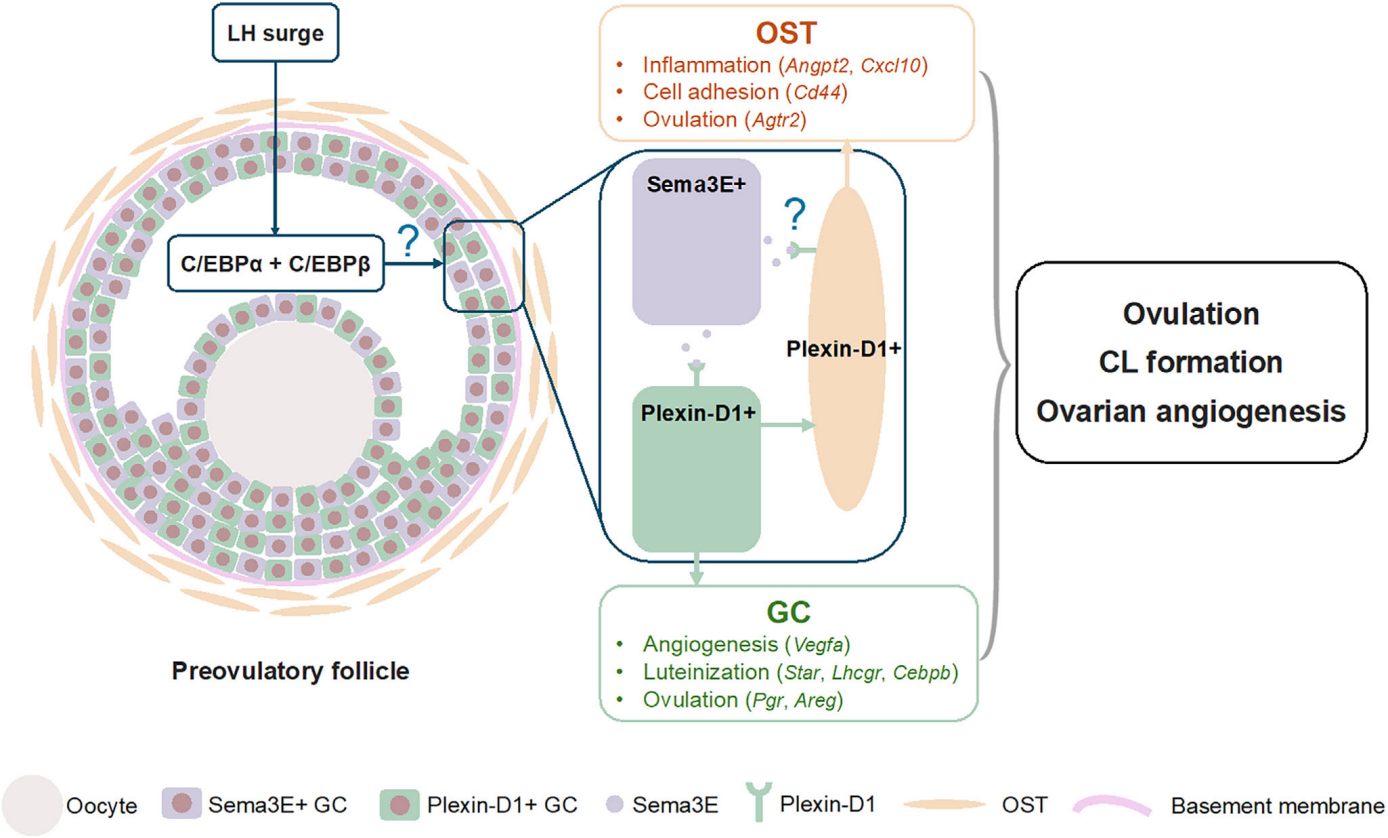
Schematic drawing of the regulation and function of Sema3E‐Plexin‐D1 pathway during ovulation. C/EBPα and C/EBPβ downstream of the LH surge are required for the normal expression of Sema3e in GCs and Plxnd1 in both GCs and OST. Sema3E, produced by *Sema3e* ^+^ GCs, acts on *Plxnd1* ^+^ GCs, thereby regulating the expression of genes controlling angiogenesis, luteinization, and ovulation. Additionally, Sema3E influences OST inflammation and cell‐cell adhesion primarily through its impact on GCs. Further investigation is warranted to elucidate how C/EBPα and C/EBPβ regulate Sema3E‐Plexin‐D1 pathway in the ovary and to determine whether and how Sema3e in GCs directly regulates OST function. GC: granulosa cell; OST: ovarian stromal tissue; CL: corpus luteum.

Transcripts of *Plxnd1* are present in various types of cells in the OST and are relatively steady except for lower levels at 0 and 11.5 h post‐hCG. The increase in *Plxnd1* transcript between 0 and 2 h post‐hCG suggests an induction by hCG, which is supported by data from in vitro cultured OST (co‐cultured with GCs) treated with For/PMA (Figure S5f, Supporting Information). In GCs and during the preovulatory stage, *Plxnd1* is mostly expressed in a small sub‐population of GCs that are identified as luteinized GCs (Figure 2f). This cluster of luteinized GCs, which exhibits high chromatin accessibility of *Plxnd1* in controls, was absent in *Cebpa/b^gc‐/‐^* mutants at both time points (Figure 2c,d)2. The deletion of *Cebpa/b* resulted in reduced chromatin accessibility at *Plxnd1* and persistently lower *Plxnd1* mRNA levels in GCs throughout the preovulatory stage (0–12 h post‐hCG) (Figure 2a,h). Given this pattern, the regulation of *Plxnd1* by C/EBPα/β in GCs may occur even earlier than the preovulatory stage, necessitating further investigation, particularly before hCG/LH stimulation. This sub‐population of luteinized GCs are sporadic and dispersed across the GC layers before ovulation (Figures 1c and 2b; Figure S2c, Supporting Information). The mRNA levels of *Plxnd1* in this sub‐population appear to be robustly regulated downstream of hCG as it is significantly induced between 0 and 6 h post‐hCG when its transcript levels are assessed in total GCs and appreciable by spatial transcriptomics (Figure 1a; Figure S1, Supporting Information). Interestingly, the luteinized GC cluster with high chromatin accessibility of *Plxnd1* also exhibits high chromatin accessibility of *Vegfa* (Figure 2f; Figure S6, Supporting Information), suggesting that despite its small number of cells, this sub‐population of GCs is important for the regulation of ovarian angiogenesis around the time of ovulation. As transcripts of *Plxnd1* are present in multiple types of cells in OST, and in luteinized GCs that are not the same population of ovulatory GCs that express *Sema3e* (Figures 2c–f and **6**), both OST and luteinized GCs can be direct targets of Sema3E produced by ovulatory GCs via paracrine mechanisms but with different downstream effects.

Our data demonstrate that the Sema3E‐Plexin‐D1 pathway is a novel regulator of ovarian angiogenesis. Sema3E has been identified as a regulator of angiogenesis in various tissues, primarily by acting on the Plexin‐D1‐expressing vascular ECs to modulate their proliferation, differentiation, adhesion, and migration. Notably, Sema3E‐Plexin‐D1 has been identified as a suppressor of the VEGFA‐induced Delta‐like 4 (Dll4)‐Notch signaling pathway, which promotes EC differentiation during vessel sprouting.^[^ ^31^ ^]^ The binding of Sema3E to Plexin‐D1 triggers intracellular events, including the inactivation of R‐Ras and activation of Arf6, which affects the adhesion of ECs to extracellular matrix (ECM), and ultimately inhibits EC migration.^[^ ^29^ ^]^ Multiple lines of evidence in this study indicate that Sema3E‐Plexin‐D1 pathway regulates ovarian angiogenesis around the time of ovulation: *i*) *Plxnd1* transcripts are expressed in *Vegfr2* ^+^ vascular ECs in the ovarian stroma and early CL; *ii*) blocking Plexin‐D1 in cultured GCs by a neutralizing antibody leads to changes in the expression of angiogenic genes; *iii*) excessive intraovarian Sema3E causes abnormal vascular structures in vivo; and *iv*) Sema3E treatment on GCs and OST in vivo and in vitro induces changes of angiogenic regulators. Sema3E produced by GCs may regulate ovarian angiogenesis around the time of ovulation through at least two mechanisms. One is by directly targeting *Plxnd1*‐expressing vascular ECs (*Vegfr2* ^+^) in the OST, the other one is by regulating the expression of angiogenic factors in a sub‐population of GCs (*Plxnd1* ^+^ luteinized GCs). We currently do not know definitively how Sema3E directly targets and regulates stromal vascular ECs. Although Sema3E produced by GCs can permeate through follicular basement membrane, which allows the exchange of low molecular weight proteins (< 500 kDa) between GCs and OST, and bind to Plexin‐D1 on OST (Figure 6), it remains challenging to isolate its direct effects. This is because it is impossible to exclude the crosstalk between GCs and OST in vivo, and in vitro culture of ovarian OST alone appears not to fully recapitulate the biology in vivo, especially regarding vascular function (Figure S5d,e Supporting Information). Furthermore, *Plxnd1* is expressed in multiple additional stromal cell types other than ECs (theca cells and interstitial cells), and Sema3E may regulate ovarian angiogenesis through a wide range of effects. For example, Sema3E‐Plexin‐D1 pathway regulates ECM proteolytic genes in the heart, contributing to the modulation of ECM remodeling.^[^ ^32^ ^]^ Such remodeling process is essential for the proper formation and stability of blood vessels during angiogenesis.^[^ ^33^ ^]^ In line with its effect on the ECM, Sema3E treatment altered the transcript levels of genes involved in ECM remodeling, including *Angpt2*, *Cxcl10*, and *Cd44* in the OST (Figures 4h and 5e).^[^ ^34^ ^]^ Furthermore, *Angpt2*, *Cxcl10*, *Selplg*, *Serpine1*, and *Cd44* also participate in the regulation of inflammation.^[^ ^24^, ^34^ ^]^ In other contexts, Sema3E‐Plexin‐D1 pathway also regulates inflammation by modulating the migration and function of immune cells,^[^ ^9^, ^35^ ^]^ which in turn plays a complex role in angiogenesis such as promoting the release of various cytokines, as well as pro‐ and anti‐angiogenic growth factors.^[^ ^36^ ^]^ Indeed, our data show that in vivo and in vitro Sema3E treatment altered the expressions of inflammatory genes in OST (Figures 4f,h and 5e).

Compelling evidence supports that Sema3E regulates OST angiogenesis by regulating the expression of angiogenic factors in *Plxnd1*+ GCs. Many studies have shown that pro‐angiogenic factors are produced by GCs under the stimulation of the LH surge and exert their effects on vascular ECs in the OST.^[^ ^11^, ^15^ ^]^ These pro‐angiogenic factors regulate the growth of new vessels from the OST into the GC layers of preovulatory follicles, which are devoid of vasculature during earlier growth and maturation stages.^[^ ^37^ ^]^ In this study, treatment of Sema3E leads to reduced expression of vascular genes in GCs in vivo and in vitro, in particular *Vegfa* (Figures 4g and 5b; Figure S5b,c, Supporting Information). This is consistent with previous reports that Sema3E overexpression leads to impaired vessel branching through downregulation of VEGFA signaling in ECs,^[^ ^31^ ^]^ and activation of Sema3E and Plexin‐D1 downregulates VEGFA signaling in the mouse brain.^[^ ^8^ ^]^ Most importantly, when cultured alone in vitro and treated with Sema3E, changes in the expression of vascular genes in OST did not mirror those in vivo unless OST are co‐cultured with GCs, indicating the important role of GC‐OST crosstalk in regulating angiogenic activities downstream of Sema3E in the OST.

The preovulatory LH surge induces in GCs the expression of genes crucial for ovulation, such as *Pgr*, *Cebpb*, and *Areg*.^[^ ^5^, ^19^, ^23^ ^]^ In this study, a notable decrease in the mRNA levels of *Pgr*, *Areg*, *Cebpb*, and key genes in GC luteinization *(Star* and *Lhcgr*) is observed in the GCs treated with Sema3E compared to controls both in vivo and in vitro (Figures 4g and 5b). This suggests Sema3E may regulate ovulation and luteinization by downregulating these critical genes in GCs. Analysis of snATAC‐seq data revealed that in normal mice at 8 h post‐hCG, the luteinized sub‐population of GCs with high chromatin accessibility of *Plxnd1* also exhibits high chromatin accessibility of *Cebpb, Star*, and *Lhcgr* (Figure 2f; Figure S6, Supporting Information). This implies potential paracrine mechanism by which Sema3E produced in ovulatory GCs acts through the *Plxnd1*+ luteinizing GCs to regulate the expression of these genes. In addition to influencing transcripts of ovulation‐ and luteinization‐related genes in GCs, treatment of Sema3E also lead to the dysregulation of factors in OST that play a role in ovulation, including increased transcripts of *Angpt2*, *Cd44*, and decreased transcripts of *Agtr2* (Figures 4h and 5e). Previous studies show that increased levels of *Angpt2* and *Cd44* are associated with reduced ovulation.^[^ ^13^, ^38^ ^]^ Specifically, intrafollicular administration of ANGPT2 reduced the rate of ovulation and elevated CD44 levels in the serum were observed in anovulatory polycystic ovary syndrome (PCOS) patients. Additionally, intrafollicular blockage of angiotensin II receptors (encoded by *Agtr2*) reduced ovulation.^[^ ^26^ ^]^ Taken together, our fundings indicate Sema3E‐Plexin‐D1 pathway regulates ovulation‐related genes in both GCs and OST.

While this study provides strong evidence for the role of Sema3E‐Plexin‐D1 pathway in preovulatory ovarian function, particularly in ovarian angiogenesis, it is not without limitations. First, the precise mechanism by which Sema3E‐Plexin‐D1 pathway regulates ovarian angiogenesis warrants further investigation. One potential mechanism is that Sema3E‐Plexin‐D1 pathway may influence ovarian angiogenesis by altering EC differentiation through the VEGFA‐induced Dll4‐Notch signaling pathway. Given that vessel sprouting is affected in ovaries treated with excessive Sema3E, it is possible that inappropriate EC differentiation underlies these vascular changes, although further studies are required to test this hypothesis. Future experiments can be conducted on isolated ovarian ECs in in vitro culture to directly test the effects of Sema3E‐Plexin‐D1 pathway; examples of such effects include the activity of Dll4‐Notch signaling, as well as EC proliferation, migration, differentiation, branching morphogenesis, and response to angiogenic regulators such as VEGFA. Furthermore, ECs‐specific *Dll4* or *Notch* knockout models, or pharmacological inhibition of Notch signaling in combination with Sema3E treatment, could provide additional mechanistic insights in the more physiological in vivo context. These studies would provide critical cellular and molecular mechanistic insights into how the Sema3E‐Plexin‐D1 pathway modulates ovarian vascular remodeling during the preovulatory period. Second, while we observed structural changes in ovarian vasculature following Sema3E treatment, the consequences of these alterations on the functionality of the ovarian vasculature remains to be tested, such as hemodynamics, changes in vascular permeability, and the subsequent tissue perfusion and metabolism. To this end, techniques such as contrast‐enhanced ultrasound or laser speckle contrast imaging can be employed in future studies. Additionally, since *Plxnd1* is expressed in multiple ovarian cell types beyond ECs, cell type‐specific gene editing approaches can be employed to disrupt Plexin‐D1 in vivo in vascular ECs or other ovarian cells to dissect cell type‐specific effects. Our data also revealed potential involvement of Sema3E‐Plexin‐D1 pathway in ovarian immune/inflammatory function as well as neuronal activities, both of which open doors to future studies. While our study established the Sema3E‐Plexin‐D1 pathway as an important regulator of ovarian function in mice, whether and how this pathway also plays a role in the human ovary remains to be tested. Several lines of evidence support conserved functions of this pathway between mice and humans, suggesting it may play a similar role in human ovarian physiology. First, both *SEMA3E* and *PLXND1* are expressed in the human ovary, with cell type‐specific expression patterns consistent with what we observed in mice:*SEMA3E* in GCs and *PLXND1* in stromal and vascular ECs.^[^ ^39^ ^]^ Second, the interaction between Sema3E and Plexin‐D1 and their downstream effects, particularly in angiogenesis, have been demonstrated in human tissues other than the ovary.^[^ ^10^, ^29^, ^40^ ^]^ Third, an elevated level of *SEMA3E* transcripts was reported in oocytes from aged women compared to younger counterparts, suggesting its involvement in ovarian health.^[^ ^41^ ^]^ However, our analysis of published transcriptomic datasets from human ovaries with pathological changes, such as PCOS, suggests dysregulation of other members in the Sema family but not *SEMA3E* itself.^[^ ^42^ ^]^ While this supports a role of semaphorins in human ovarian pathology, future studies are needed to zoom in on the preovulatory period when the activity of the Sema3E‐Plexin‐D1 pathway is dynamically regulated as we have shown in mice. Taken together, our data highlight the need to further investigate the Sema3E‐Plexin‐D1 pathway in the health and diseases of the human ovary as a regulator of ovarian function and a potential therapeutic target.

In conclusion, our data demonstrate the importance of Sema3E‐Plexin‐D1 pathway in ovarian function, highlighting its role in regulating ovulation, GC luteinization, ovarian angiogenesis, and inflammation (Figure 6). This knowledge opens potential new therapeutic avenues for treating ovarian dysfunction and infertility in women.

## 4 Experimental Section

### Animals

C57BL/6J mice were bred in‐house for all experiments in this study. *Cebpa/b^fl/fl^;Cyp19a1‐Cre* mutant mice were generated as previously described.^[^ ^5^ ^]^ The *Cebpa/b^fl/fl^* mice (*Cebpa/b^gc+/+^* mice, without *Cyp19a1‐Cre*) were used as controls for *Cebpa/b^gc‐/‐^* mutant mice. Mice were housed in 11.5″ x 7.5″ IVC Polycarp Shoebox Cage for the duration of the experiment. Temperature 68–77°F and humidity between 30 and 70% were maintained in the rodent room. Lights were turned ON from 5 AM and OFF at 7 PM in the rodent room. All animal work was conducted ethically, conforming to the U.S. Public Health Service policy, and was approved by the Institutional Animal Care and Use Committee at Cornell University (IACUC approved protocol number 2019‐0006).

### Superovulation

Around 21–23 days old female mice (immature) weighing 10∼11 g were injected intraperitoneally with 5 IU pregnant mare serum gonadotropin (PMSG, NATE‐0969, Creative Enzymes) followed 44–48 h later with the injection of 5 IU human chorionic gonadotropin (hCG, 9002‐61‐3, Sigma Aldrich). Granulosa cells (GCs) were isolated by needle puncture from ovaries, with the residual ovarian tissues collected as ovarian stromal tissues (OSTs). GCs, OST, or whole ovaries were collected from superovulated mice for subsequent analyses. To assess the rate of ovulation, cumulus‐oocyte complexes (COCs) of superovulated mice were collected from both oviducts and counted at 24 h post‐hCG.

### RNA Extraction, Reverse Transcription, and Real‐Time Quantitative PCR (RT‐qPCR)

Total messenger RNA (mRNA) was extracted using RNeasy Micro Kit (74106, Qiagen). Reverse transcription was performed using a High‐Capacity cDNA Reverse Transcription Kit (4368814, Applied Biosystems). RT‐qPCR was performed using RT^2^ SYBR Green ROX FAST Mastermix (330623, Qiagen) on StepOnePlus Real‐Time PCR System (4376600, Applied Biosystems). Relative levels of mRNA were calculated using the 2^(‐ΔCt)^ method (2^(‐ΔΔCt)^ method in Figure 4g,h) and normalized using housekeeping gene *Rpl19*. The primers used are listed in Table S3 (Supporting Information).

### Fluorescence In Situ Hybridization (FISH)

Fresh ovaries were collected from WT, *Cebpa/b^gc+/+^*, and *Cebpa/b^gc‐/‐^* mice at 8 h and 24 h post‐hCG, immediately embedded in Optimal Cutting Compound media (4583, SAKURA), and later sliced into 25 µm thick sections for FISH. Digoxigenin (DIG)‐labeled mRNA antisense probes against *Plxnd1* and fluorescein (FITC)‐labeled mRNA antisense probes against *Vegfr2* were generated using reverse‐transcribed mouse cDNA as a template and RNA DIG‐ or FITC‐labeling kits from Roche (Sigma). Primer and probe sequences are from genepaint.org for the*Vegfr2* and from Allen Brain Atlas for *Plxnd1* (Table S4, Supporting Information). FISH was performed by the RNA In Situ Hybridization Core at Baylor College of Medicine using an automated robotic platform as previously described with modifications of the protocol for double FISH.^[^ ^43^ ^]^ In brief, both probes were hybridized to the tissue sections simultaneously. After the described washing and blocking steps, the DIG‐labeled probes were visualized using tyramide‐Cy3 Plus (1/50 dilution, 15‐min incubation, Akoya). After washing in Tris‐NaCL‐Tween buffer (TNT), the remaining HRP‐activity was quenched by a 10‐min incubation in 0.2 M HCl. The sections were then washed in TNT, blocked in Tris‐NaCl‐blocking buffer (TNB) for 15 min before a 30‐min incubation at room temperature with HRP‐labeled sheep anti‐FITC antibody (1/500 in TNB, Roche). After washing in TNT, the FITC‐labeled probe was visualized using tyramide‐FITC Plus (1/50 dilution, 15‐min incubation, Akoya). Following washing in TNT the slides were stained with DAPI (Invitrogen), washed again, removed from the machine, and mounted in ProLong Diamond Antifade Mountant (Invitrogen). Imaging was performed at the Integrated Microscopy Core at BCM on the GE Healthcare DVLive epifluorescence image restoration microscope. FISH expression was quantified using ImageJ (Fiji, Version: 2.9.0). For quantifications data shown in Figure S2d (Supporting Information), *Plxnd1‐ and Vegfr2‐* positive areas were normalized to the DAPI‐positive area of the whole ovaries. Colocalization analysis of *Plxnd1* with *Vegfr2* was performed using the Coloc 2 Plugin.

### Immunofluorescence (IF)

Ovaries were fixed in 4% paraformaldehyde (15710, Electron Microscopy Sciences) for 4 h at 4 °C, then washed with phosphate buffered saline (PBS, QB‐119‐069‐491, Neta Scientific) and stored in 75% ethanol (64‐17‐5, Fisher Scientific) before being dehydrated and embedded in paraffin. For immunofluorescent staining, paraffin sections of 5 µm thickness were deparaffinized and autoclaved in citric acid buffer (pH 6.0). Sections were blocked with 2% bovine serum albumin (BSA, BP9700100, Fisher Scientific)‐PBS containing 0.1% Tween 20 for 1 h at room temperature and then incubated with primary antibody (anti‐Sema3E, AF3239, R&D at 1:100) overnight at 4 °C. Sections were washed and incubated with secondary antibody (Goat Anti‐Rabbit IgG (H+L), A‐11012, Invitrogen) conjugated with Alexa 594 at 1:1000 dilution for 1 h at room temperature, followed by cell nucleus counterstaining with 1 µg mL^−1^ Hoechst 33 342 (76482‐876, VWR). Images were obtained using Nikon Diaphot 300 microscope (Nikon Instruments) and processed with ImageJ. Signals of Sema3E staining were quantified using ImageJ. The area of preovulatory follicle was drawn manually. The areas of Sema3E staining were normalized to the Hoechst‐positive areas of preovulatory follicles.

### Intraovarian Injection of Recombinant Sema3E

Under anesthesia by 2% of isoflurane, the skin of the dorsal lumbar region of mice was shaved to expose the surgical field where a small incision was made. Ovaries were located by the ovarian fat pad underneath the peritoneal wall, which was cut open to expose the ovary. One ovary was injected with 2 µg of Sema3E (mouse recombinant protein, 3238‐S3, R&D) in 10 µL of PBS (0.2 µg µL^−1^) supplemented with 0.1% BSA (Sema3E group). The contralateral ovary was injected with 10 µL of PBS with 0.1% BSA or 2 µg of IgG (IgG group) (mouse IgG, AF007, R&D) in 10 µL of PBS with 0.1% BSA (control group). The injection was performed using a Guage 30 needle (8881250032, Monoject) to ensure precision. After each injection, the needle was gradually removed to prevent potential leakage. After injection, the incision was sutured, and mice were placed on a heating pad for recovery until awake and active. After the surgery, mice were returned to their cages to recover before subsequent experiments.

### Whole‐Mount Staining and Imaging

After mice were under anesthesia by isoflurane, the ovarian vasculature was labeled by retro‐orbital injection of 10 µL DyLight 649 labeled Lycopersicon Esculentum (Tomato) Lectin (LEL, TL) (L32472, ThermoFisher). Mice were euthanized by CO_2_ inhalation for 5 min after injection. Ovaries were dissected and fixed in 4% paraformaldehyde at 4 °C for 4 h. Fixed ovaries were then washed three times in PBS for 10 min each before clearing. The CUBIC1/2 solutions were made as previously described.^[^ ^44^ ^]^ Ovaries were cleared in CUBIC‐1 solution with gentle rocking at room temperature for 4–5 days. After washing three times in PBS for 10 min each, ovaries were incubated in PBS with 20% sucrose overnight. Subsequently, cell nuclei were stained with Hoechst 33258 in CUBIC‐2 solution for at least 24 h with gentle rocking at room temperature. Lastly, ovaries were washed using CUBIC‐2 solution for 4 h and stored in fresh CUBIC‐2 solution at room temperature until further imaging.

Ovaries were imaged using an inverted laser scanning confocal microscope (Zeiss LSM880 microscope). Images were taken at 10 µm intervals in z‐stack starting from the outer surface of the ovary to generate a 550 to 600 µm‐thick z‐stack. Z‐stack images were analyzed and transformed into 3D projection images using ImageJ. Different CL/F structures were manually defined from z‐stack images and counted as described above. Quantification of vascular density was performed using ImageJ: the areas of CL were drawn manually and lectin signals were normalized to the area of CL.

### Hessian Tubeness Analysis

Features of ovarian vasculature were analyzed and quantified using *Hessian Tubeness* filters[20] at defined widths (s = 3, 9, and 27 µm), resulting in image stacks of vascular tubes segmented by these different sizes. The filter sizes were chosen based on typical vessel diameters: capillaries range from 4 to 10 µm,^[^ ^45^ ^]^ while the average vessel diameter in ovarian stromal tissue is ≈20 µm.^[^ ^46^ ^]^ To account for the abnormally thick vessels observed in the stromal tissue of Sema3E‐injected ovaries, a filter size of 27 µm was included to ensure their detection in the treated group. Relative volume (RV) is defined as the total volume of vascular tubes at each size (*Otsu* segmented)^[^ ^47^ ^]^ normalized to the total volume of the corresponding ovary (*Triangle* segmented)^[^ ^48^ ^]^. Average intensity (AI) is defined as the average pixel value in the Tubeness‐filtered, Otsu‐segmented datasets. Total Intensity (TI) is calculated by: TI = RV*AI. All analyses were carried out using scripts written and executed using the macro scripting capabilities within the Fiji version of ImageJ (available upon request).

As vasculature becomes more complex, such as via branching morphogenesis, it will exhibit a higher surface‐area (SA) to volume (V) ratio. To demonstrate this ratio in a dimensionless quantity, the surface area must be normalized to volume units (3D rather than 2D).  *Sphericity* (ψ) is given by:^[^ ^49^ ^]^

(1) $$\psi =\pi^{\frac{1}{3}}{\left(6V\right)}^{\frac{2}{3}}/SA$$

In order to express a quantity that increases with vascular complexity, we define the dimensionless surface‐area to volume ratio (DSAV) given by:

(2) $$DSAV=\psi^{-3/2}={\left(SA\right)}^{\frac{3}{2}}/6V\pi^{1/2}$$

which is normalized to 1 for a sphere. To determine this ratio, a 3D mesh and its surface area were calculated for each dataset using the IDL image analysis and visualization environment (L3Harris Geospatial, script available upon request).

### Bulk RNA‐Sequencing (RNA‐seq) and Data Analysis

Total mRNA was extracted from GCs and OSTs as described above using the RNeasy Micro Kit according to the manufacturer's instructions. GCs or OSTs isolated from one ovary were used as one replicate, and three independent biological replicates were used for RNA‐seq library generation. Library preparation and transcriptome sequencing were conducted by Novogene Co., LTD (Davis, USA). Raw RNA‐seq reads were aligned to the mouse (GRCm38/mm10) genome using HISAT2 (version 2.0.5) by default parameters. Raw read counts for each gene were generated using featureCounts (v1.5.0‐p3) and Transcripts Per Million (TPM) values of each gene were generated using EdgeR (version 1.3.3). Only genes expressed in at least one replicate with TPM >1 were included for further bioinformatics analyses. DESeq2 R package (version 1.20.0) was used for differential gene expression analyses with cutoffs absolute FoldChange > 2 and *P*‐values < 0.05. Differentially expressed genes (DEGs) were visualized using VolcaNoseR.^[^ ^50^ ^]^ Gene Ontology (GO) analyses were performed using the online tool Metascape (http://metascape.org)^[^ ^51^ ^]^ and visualized using Hiplot (ORG) (https://hiplot.org).^[^ ^52^ ^]^

### Isolation and In Vitro Culture of Primary Granulosa Cells and Ovarian Stromal Tissues

Ovaries were collected from PMSG‐primed (24 h) 21–23 days old female mice. Primary GCs were isolated from ovaries by repetitive needle puncture. GCs and the residual OSTs were cultured in Dulbecco‐modified Eagle medium nutrient mixture F‐12 (DMEM/F12) (111330‐032, Gibco) supplemented with penicillin‐streptomycin (15 140 122, Gibco), and 5% fetal bovine serum (FBS) (35‐015‐CV, Corning). All cells/tissues were maintained in a humidified incubator in 5% CO_2_ at 37 °C. After overnight culture, GCs or OSTs were treated with forskolin (J63292‐MA, ThermoFisher) and phorbol 12‐myristate 13‐acetate (PMA, P1585, Sigma) (For/PMA) to mimic the effect of LH surge in vitro.^[^ ^53^ ^]^ Sema3E (recombinant mouse protein, 3238‐S3, R&D) or a neutralizing antibody of Plexin‐D1 (human, AF4160, R&D) was added to the cell culture medium at the same time as For/PMA. GCs or OSTs were harvested for RNA extraction at 0 h (right before For/PMA treatment), or at 24 h and 48 h post‐For/PMA.

In co‐culture experiments, GCs and OSTs were first cultured separately in 5% FBS DMEM/F12 overnight. After overnight culture, OSTs were transferred to the well with the attached GCs and treated with For/PMA and Sema3E. OSTs were harvested for RNA extraction at 24 and 48 h post‐For/PMA.

### Western Blotting (WB)

Granulosa cells were lysed in RIPA Lysis buffer (BP‐115, Boston BioProducts). Proteins of different molecular weights were separated using a 10% Bis‐Tris gel and transferred to an immobilon‐P membrane (Millipore). Membranes were blocked with 3% BSA‐TBST for 1 h at room temperature and then incubated with primary antibodies (VEGFA at 1:100 dilution, Proteintech, 19002‐1‐AP; C/EBPβ at 1:1000 dilution, Thermo Fisher Scientific, MA1‐827) overnight at 4 °C. Membranes were washed and incubated with corresponding secondary antibodies (Goat anti‐Rabbit IgG (H+L) HRP, Thermo Fisher Scientific, 31460; Goat anti‐mouse IgG (H+L) HRP, Thermo Fisher Scientific, 32430) at 1:5000 dilution for 1 h at room temperature. Images were taken using laser fluorescence scanner (LI‐COR), processed and quantified with ImageJ.

### Single‐Nucleus Assay for Transposase‐Accessible Chromatin Sequencing (snATAC‐seq)

snATAC‐seq was conducted following a previously described protocol with some modifications for ovarian tissues.^[^ ^54^ ^]^

Expression and Purification of Homemade Tn5: We utilized an adapted protocol based on Hennig B.P.'s method for homemade Tn5.^[^ ^55^ ^]^ The Tn5 cassette, PCR‐amplified from pTXB1‐Tn5, was subsequently subcloned into the pET‐SUMO vector with the following orientation: 5′ BamHI (ATG) – Tn5 – (TAA) HindIII 3′. The resulting pET‐SUMO‐Tn5 plasmid was transformed into BL21 Star (DE3) pLysS E. coli. Cells were cultured in TB/LB (1:1) supplemented with kanamycin and chloramphenicol at 37 °C until reaching OD600 ≈0.5. Subsequently, the temperature was reduced to 18–20 °C and expression of Tn5 was induced with 0.2 mM IPTG. After overnight growth at 18 °C‐20 °C, cells were harvested by centrifugation. The cell pellets were resuspended in binding buffer (20 mM HEPES‐NaOH pH 7.2, 800 mM NaCl, 20 mM imidazole, 0.2 mM EDTA, 2 mM DTT, 10% glycerol, and 0.2% Triton‐X100) with cOmplete protease inhibitors and lysed by sonication. The insoluble debris was pelleted by centrifuging the sonicated bacteria at 16000 g at 4 °C for 30 min. Polyethyleneimine (PEI) pH 7.2 was added dropwise to a final concentration of 1% for nucleic acid removal. Centrifuge at 16000 g at 4 °C for 30 min. The supernatant was incubated with 2 ml Ni‐charged Profinity IMAC Resin for 1 h with shaking. The mixture was loaded onto 10 mL Pierce Centrifuge Columns for gravity flow‐through. The mixture was washed with 8 mL of binding buffer for 5 times. The His‐SUMO‐Tn5 was eluted in 6 mL binding buffer containing 300 mM imidazole, followed by an additional 6 ml of binding buffer. To remove the fusion tag, homemade His‐tagged‐ulp‐1 proteinase was added to the elution fractions. The samples were loaded into Slide‐A‐Lyzer G2 Dialysis Cassettes (10K MWCO, 15 mL) and digested overnight at 4 °C while being dialyzed back to the binding buffer. To eliminate His‐SUMO and His‐ulp‐1, the dialyzed sample was bound to 2 mLNi‐charged Profinity IMAC Resin, loaded onto 10 mL Pierce Centrifuge Columns, and the untagged Tn5 was collected in the flow‐through by gravity. Concentration and buffer exchange were performed using Amicon Ultra‐15 Centrifugal Filter Units (30K) and transitioned to the storage buffer (100 mM HEPES‐KOH at pH 7.2, 0.2 M NaCl, 0.2 mM EDTA, 2 mM DTT, 0.2% Triton X‐100, 20% glycerol).^[^ ^56^ ^]^ The concentration was determined using the Pierce 660 nm Protein Assay. The Tn5 concentration was adjusted to 5 µM using the storage buffer and stored at ‐80 °C.

Transposome Assembly: Tn5 transposase (5 µM) was diluted by adding 1 volume of 80% glycerol, resulting in a concentration of 2.5 µM. Subsequently, Tn5 transposome was assembled by adding 0.1 volume of Tn5 adaptors (25 µM) to the Tn5 (2.5 µM) and the mixture was incubated at room temperature overnight. The assumed concentration of the transposomes was 2 µM.

Tagmentation and Sample Processing: The nuclei extraction protocol employed the same lysis buffer (1x Homogenization Buffer) and 1x washing buffer (1x ATAC‐RSB + 0.1% Tween‐20), as described by *Corces, M*.^[^ ^57^ ^]^ The modification in the extraction steps includes two homogenization stages: initially in 1x Homogenization Buffer (HB) with a loose pestle, followed by the second homogenization step in 1x Washing buffer. Nuclei suspension (8 µL) was distributed onto a 96‐well plate and 3 µL of two barcoded transposomes were added to each well. Each well contains ≈5000 nuclei and 400 µM transposomes in 1X TD buffer (10 mM Tris‐HCl, pH 7.6, 5 mM MgCl2, 10% DMF, 0.33X PBS, 0.1% Tween20, 0.01% Digitonin). The plate was incubated for 30 min at 50 °C and the reaction was terminated by adding 10 µL 40 mM EDTA to each well. All nuclei were combined into a single sample and 2 mL of Sorting buffer (1 X PBS, 0.2% BSA, 2 mM EDTA) was added. Intact nuclei were then stained with 20 µL of Draq7 (ab109202, abcam) on ice for 15 min, filtered through a 30 µm filter, and sorted by FACS using a Cell Sorter (BD FACSMelody). 25 nuclei were distributed into each well of a 96‐well plate pre‐loaded with 10 µL Sort EB (10 mM Tris‐HCl, pH 8.0, 0.05% SDS), and incubated for 15 min at 50 °C to lysis nuclei and finish tagmentation. 2.5 µL of 5% Triton‐X100 was added into each well to quench SDS prior to PCR.

PCR Amplification (Library Generation) and Size Selection: For each well in a 96‐well plate, the 12.5 µL of 2X PCR reaction master mix contains 5 µL Q5 Reaction buffer, 5 µL High GC Enhancer, 0.5 µL dNTP mix (10 mM), 0.25 µL Q5 DNA Polymerase, 0.5 µL Universal P5 primer (25 µM), 0.75 µL Nuclease‐Free Water, and a barcoded i7 primer were added. Libraries were amplified using the following program: 72 °C for 5 min, 98 °C for 30 sec, then 14 cycles of (98 °C for 15 sec, 66 °C for 30 sec, 72 °C for 40 sec). PCR products from all wells were repooled and purified using QIAquick spin columns, followed by two rounds of size selection with AMPure XP beads. The first round is at 0.5 X/1.2 X and the second round is at 1.2 X. Finally, the DNA was eluted in 40 µL of 10 mM Tris, pH 8.0.

Library Quantification, Quality Control, and Sequencing: The concentration of libraries was measured using the Qubit. DNA fragment length distribution was analyzed by Bioanalyzer High Sensitivity DNA Chip (Agilent Genomics). Libraries were sequenced using the Illumina HiSeq X platform.

### snATAC‐seq Data Pre‐Processing and Analysis

Undemultiplexed fastq files were processed using cutadapt,^[^ ^58^ ^]^ UMI tools,^[^ ^59^ ^]^ and custom scripts^[^ ^54^ ^]^ to parse and assemble combinatorial barcodes from read segments and extract valid single‐nucleus barcodes (with error correction). Adapter sequences were trimmed using cutadapt (version 4.1). Alignment to the mm10 reference genome was done using bowtie2. Mitochondrial reads and blacklisted regions were then removed. Duplicate reads were removed using umi_tools dedup (version 1.1.4). Identification of cell clusters and cell types were performed in ArchR.^[^ ^60^ ^]^ We initially obtained 13347 cells after filtering out cells that had less than 1000 fragments and a transcription start site (TSS) enrichment score of less than 4. After filtering out doublets, there were 13095 cells with a median TSS score of 13.376 and a median number of fragments of 9040. After the initial quality check, IterativeLSI (Latent Semantic Indexing) was added to the ArchR project. The data was then clustered using the default resolution of 0.8. The clusters were visualized using uniform manifold approximation and projection (UMAP). A total of 13 clusters were identified and cell types were assigned using marker genes from the current literature.^[^ ^18^, ^19^ ^]^ To visualize genes of interest, gene scores were imputed using MAGIC. Genes of interest were then visualized on the UMAP. To visualize co‐accessibility, peaks were first called using MACS2 and a peak matrix was added to the ArchR project. Marker peaks were identified, and co‐accessibility was calculated using the “addCoAccessibility” function. Browser tracks were plotted for the clusters of interest (clusters 2, 5, 6) for the genes *Sema3e* and *Plxnd1*.

### Statistical Analysis

All quantitative data are presented as mean ± standard deviation (SD). Statistical analysis was performed using the GraphPad Prism 10 analysis software. Each experiment included at least three independent biological samples and was repeated at least three times. Sample sizes, the statistical tests used, and *P*‐values were described in each figure legend. Cross‐sample comparisons were made using two‐tailed unpaired Student's *t*‐test between two groups. For comparison across three or more groups, One‐way or two‐way ANOVA followed by Tukey's multiple comparisons test was used. *p‐*value < 0.05 was considered statistically significant. ^*^ *p* < 0.05; ^**^ *p* < 0.01; ^***^ *p* < 0.001.

## Conflict of Interest

The authors declare no conflict of interest.

## Author Contributions

H.Z. and Y.A.R. designed the study. H.Z. performed the experiments and analyzed the data. M.C.L. performed FISH. R.B.W. performed Hessian Tubeness analysis. J.K.G. developed the method for snATAC‐seq. B.S. performed library preparation for snATAC‐seq. P.D.S., P.R.M, S.L. and J.D. analyzed the data from snATAC‐seq. R.B.L. provided feedback on Bulk RNA analysis. Y.L analyzed the publicly available single‐cell RNA seq data. H.Z. and Y.A.R. wrote the manuscript. All authors provided feedback and comments.

## Supporting information



Supporting Information

Supplemental Table 1

Supplemental Table 2

## Data Availability

The Bulk RNA sequencing data that support the findings of this study are openly available in NCBI's Gene Expression Omnibus at https://www.ncbi.nlm.nih.gov/geo/, reference number 262209. The snATAC sequencing data that support the finding of this study are available from the corresponding author upon reasonable request.
