# Supplementary material for: Semaphorin 3E‐Plexin‐D1 Pathway Downstream of the Luteinizing Hormone Surge Regulates Ovulation, Granulosa Cell Luteinization, and Ovarian Angiogenesis in Mice
Source: Adv Sci (Weinh). 2025 May 20;12(29):e17163. doi: 10.1002/advs.202417163 (PMC12362816; doi:10.1002/advs.202417163)
Supplement: Supplementary file 1 — Supporting Information [file ADVS-12-e17163-s003.docx]

**Supporting Information**

Semaphorin 3E-Plexin-D1 pathway downstream of the luteinizing hormone surge regulates ovulation, granulosa cell luteinization, and ovarian angiogenesis in mice

**Figure S1.**

**a)** Spatial transcriptomics maps of the Mantri et al. dataset (GSE240271) showing the expression of *Sema3e* and *Plxnd1* over eight time points before and after hCG stimulation in the immature mouse ovary.

**b)** Single-cell RNA seq UMAP plot of the Morris et al. dataset (SCP1914) showing the expression of *Sema3e* and *Plxnd1* in adult cycling mouse ovary.

**
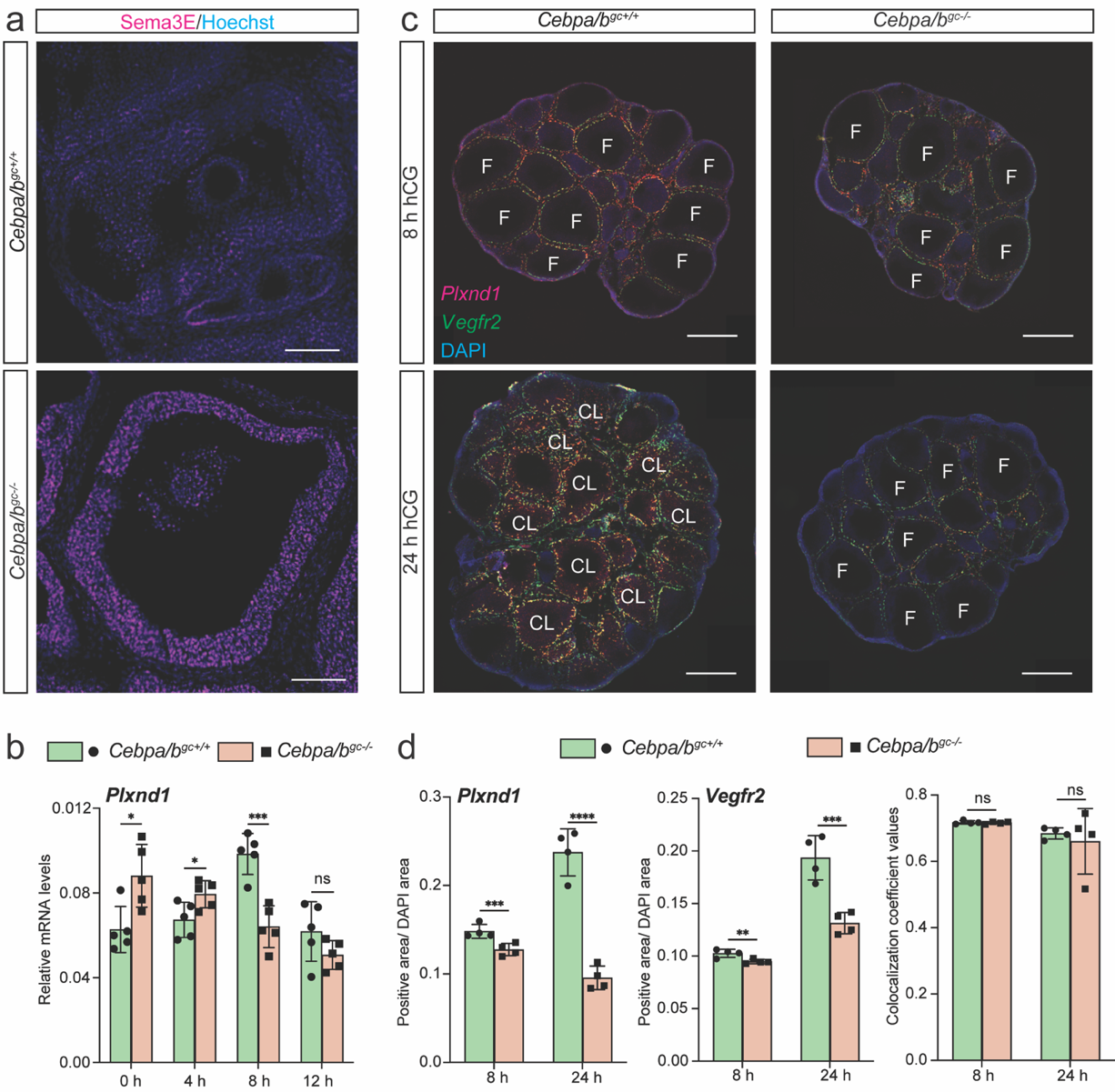
**

**Figure S2. Granulosa cell-specific deletion of *Cebpa* and *Cebpb* leads to dysregulated expression of *Sema3e* and *Plxnd1* in the ovary.**

a) Representative images of IF staining of Sema3E in ovaries from *Cebpa/b^gc+/+^* and *Cebpa/b^gc-/-^* mice at 8 h post-hCG. Scale bar = 100 μm. b) RT-qPCR shows the mRNA levels of *Plxnd1* in OSTs of *Cebpa/b^gc+/+^* (n=5) and *Cebpa/b^gc-/-^* mice (n=5) before and after hCG treatment. c) Representative images of FISH of *Plxnd1* (red) and *Vegfr2* (green) mRNA in ovaries of *Cebpa/b^gc+/+^* (n=4) and *Cebpa/b^gc-/-^* mice (n=4) at 8 h and 24 h post-hCG treatment. Scale bar = 500 μm. d) Quantification and colocalization analysis of FISH in c. FISH signals were quantified by normalizing the *Plxnd1*- or *Vegfr2*-positive area to the DAPI-positive area. Quantitative data are presented as mean ± SD. Multiple two-tailed unpaired student’s test was used for statistical analysis in b and d. **p*< 0.05; ***p* < 0.01; ****p*< 0.001; ns, not significance. F: follicle; CL: corpus luteum.

**
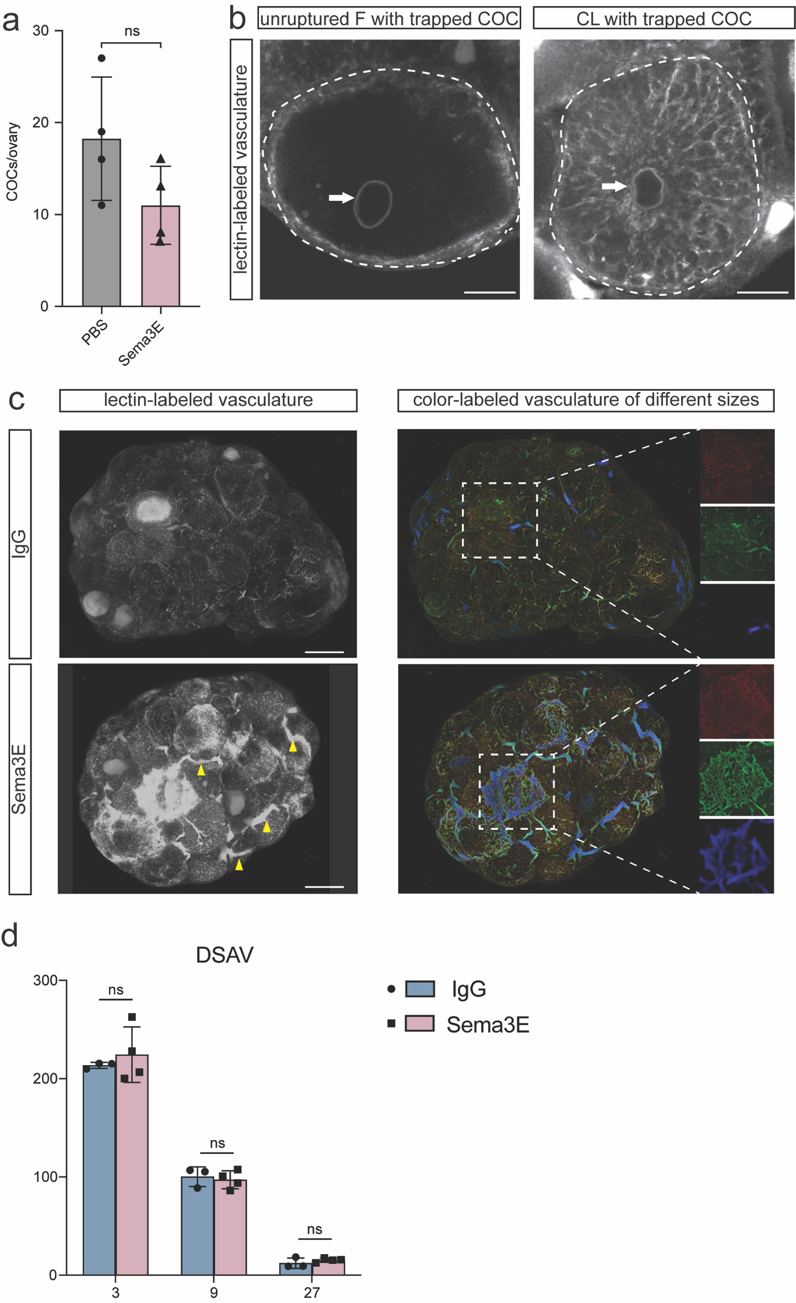
**

**Figure S3.** **Excessive Sema3E reduces ovulation rate and affects ovarian vasculature**.

a) The numbers of COCs from ovaries injected with Sema3E (n=4) and PBS (n=4), respectively, at 8 h post-hCG. b) Representative cross-section images of different structures from ovaries shown in Figure 2c. Left: unruptured F with trapped COCs; right: CL with trapped COCs. F/CL is outlined by white dashed lines. Arrow: oocyte. Scale bar = 100 μm. c) Representative whole-mount 3D projection images of cleared ovaries in Figure 2c, with vessels of different sizes labeled with different colors. Red: 3 μm; green: 9 μm; Blue: 27 μm. Scale bar = 500 μm. d) Hessian tubeness analysis on Dimensionless Surface Area to Volume Ratio (DSAV) of images in Figure 2c. Quantitative data were all presented as mean ± SD. Two-tailed unpaired student’s test was used for statistical analysis in a. Multiple two-tailed unpaired student’s test was used for statistical analysis in c. **p* < 0.05; ***p* < 0.01; ****p* < 0.001; ns, not significance. COC: cumulus-oocyte complex; F: follicle; CL: corpus luteum.

**
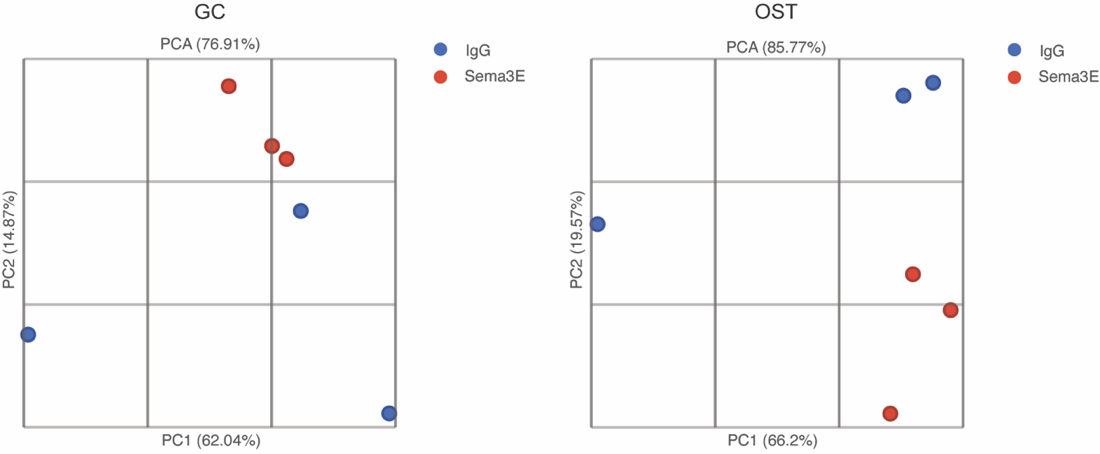
**

**Figure S4.** Principal component analysis (PCA) of samples from GCs and OSTs of IgG- and Sema3E-injected ovaries. GC: granulosa cell; OST: ovarian stromal tissue.

**
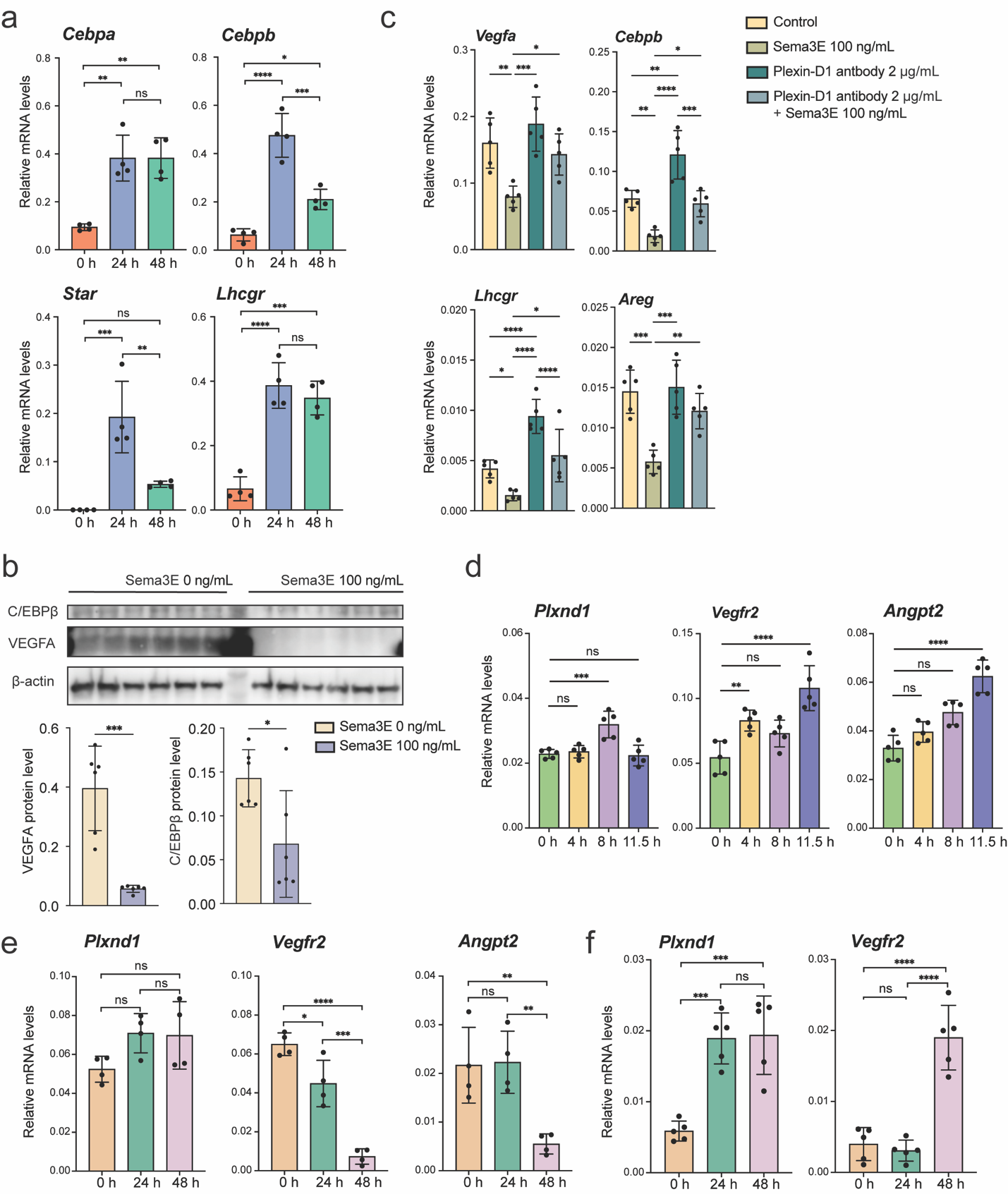
**

**Figure S5.  Crosstalk between granulosa cells and the ovarian stromal tissue is crucial for stromal angiogenesis and inflammation。**

a) RT-qPCR shows the induction of mRNA of luteinization-related genes in in vitro cultured GCs (n=4) after For/PMA treatment. b) Western blot shows VEGFA and C/EBPβ protein levels in in vitro cultured GCs (n=6) treated with or without recombinant Sema3E at 24 h post-For/PMA-induce luteinization. c) RT-qPCR shows the mRNA of selected genes in in vitro cultured GCs (n=5) at 24 h post-For/PMA-induce luteinization. GCs were pre-treated with Plexin-D1 neutralizing antibody or vehicle for 1 h followed by Sema3E treatment. d) RT-qPCR shows the in vivo mRNA levels of selected genes in OSTs (n=5) before and after hCG treatment. e) RT-qPCR shows the mRNA levels of selected genes in in vitro cultured OSTs (n=4) before and after For/PMA treatment. f) RT-qPCR shows the mRNA levels of selected genes before and after For/PMA treatment in in vitro cultured OSTs (n=5) that were co-cultured with GCs. Quantitative data were all presented as mean ± SD. One way ANOVA with Tukey’s multiple comparisons test was used for statistical analysis in a-d. **P* < 0.05; ***P* < 0.01; ****P* < 0.001; ns, not significance. GC: granulosa cell; OST: ovarian stromal tissue.


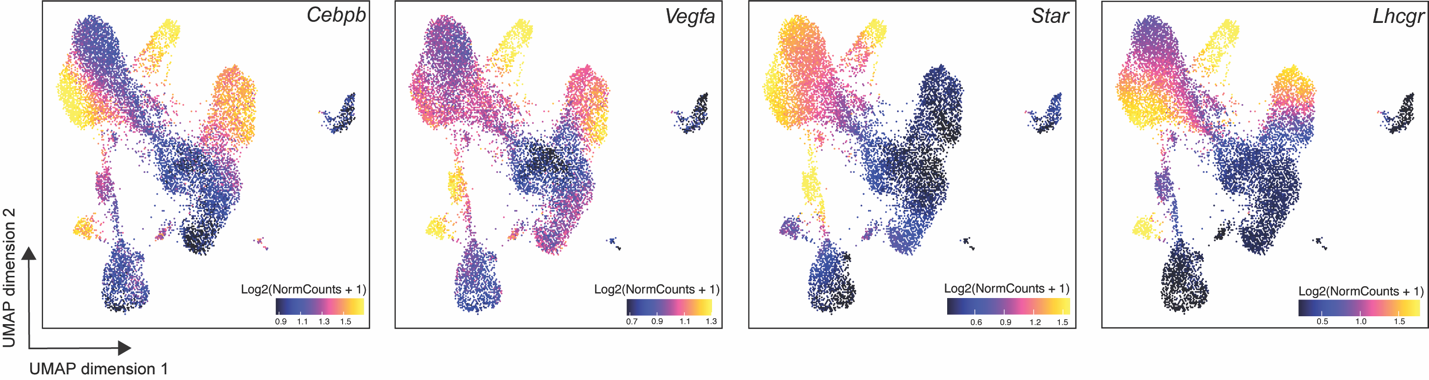


**Figure S6.**

UMAP visualization colored by log2 normalized gene scores demonstrating the chromatin accessibility of selected gene in clusters illustrated in Figure 2c and 2d. Gene scores are calculated as Log2(NormCounts + 1).

**Table S3.** Primers Used for RT-qPCR.

| Genes | Forward primer sequence | Reverse primer sequence |
| --- | --- | --- |
| *L19* | GGTGACCTGGATGAGAAGGA | TTCAGCTTGTGGATGTGCTC |
| *Sema3e* | TTTCAAGGGCTTGGATACAACTC | CAGTTATACCCTGGGAACCTGT |
| *Plxnd1* | CGCAACCGTAGCCTAGAAGAC | GGTTAAGGTCGAAGGTGAAGAG |
| *Vegfa* | CCCACGACAGAAGGAGAGCAGAAGT | CATCAGCGGCACACAGGACGG |
| *Pgr* | CTCCGGGACCGAACAGAGT | ACAACAACCCTTTGGTAGCAG |
| *Areg* | AGTGCTGTTGCTGCTGGTC | ACTGTGGTCCCCAGAAAGC |
| *Cebpb* | CAAGCTGAGCGACGAGTACA | AGCTGCTCCACCTTCTTCTG |
| *Star* | CATTGGCCAAGAGCTCAACT | ACCTCTCCCTGCTGGATGTA |
| *Lhcgr* | CTGAAAACTCTGCCCTCCAG | AATCGTAATCCCAGCCACTG |
| *Eng* | CCTTTGGTCGGCATGGTAG | GTCCCCTCTGACCCAAGGTT |
| *Dll4* | TTCCAGGCAACCTTCTCCGA | ACTGCCGCTATTCTTGTCCC |
| *Nrarp* | AAGCTGTTGGTCAAGTTCGGA | CGCACACCGAGGTAGTTGG |
| *C5ar1* | ATGGACCCCATAGATAACAGCA | GAGTAGATGATAAGGGCTGCAAC |
| *Angpt2* | CCTCGACTACGACGACTCAGT | TCTGCACCACATTCTGTTGGA |
| *Cd44* | TCTGCCATCTAGCACTAAGAGC | GTCTGGGTATTGAAAGGTGTAGC |
| *Cxcl10* | CCAAGTGCTGCCGTCATTTTC | GGCTCGCAGGGATGATTTCAA |
| *Agtr2* | ATGATTGGCTTTTTGGACCTGT | AAGGGTAGATGACCGATTGGT |
| *Selplg* | GTCTGTCCCGTCACTGGATAC | GTCTGTCCCGTCACTGGATAC |
| *Serpine1* | TTCAGCCCTTGCTTGCCTC | TTCAGCCCTTGCTTGCCTC |
| *Wnt4* | AGACGTGCGAGAAACTCAAAG | GGAACTGGTATTGGCACTCCT |

**Table S4.** Probes Used for FISH.

| Genes | Forward primer sequence | Reverse primer sequence |
| --- | --- | --- |
| *Plxnd1* | TAGAGATCCAGCGCCGTTTC | AGCGGTGGTCTTCTAGGCTA |
| *Vegfr2* | ATCTTTGGTGGAAGCCACAG | CATGCGCTCTAGGATGATGA |
